# Multi-Faceted Proteomic Characterization of Host Protein Complement of Rift Valley Fever Virus Virions and Identification of Specific Heat Shock Proteins, Including HSP90, as Important Viral Host Factors

**DOI:** 10.1371/journal.pone.0093483

**Published:** 2014-05-08

**Authors:** Jonathan E. Nuss, Kylene Kehn-Hall, Ashwini Benedict, Julie Costantino, Michael Ward, Brian D. Peyser, Cary J. Retterer, Lyal E. Tressler, Laura M. Wanner, Hugh F. McGovern, Anum Zaidi, Scott M. Anthony, Krishna P. Kota, Sina Bavari, Ramin M. Hakami

**Affiliations:** 1 US Army Medical Research Institute of Infectious Diseases, Frederick, Maryland, United States of America; 2 School of Systems Biology, and National Center for Biodefense & Infectious Diseases, George Mason University, Manassas, Virginia, United States of America; 3 University of Texas Health Sciences Center, Houston, Texas, United States of America; The University of Texas Medical Branch, United States of America

## Abstract

Rift Valley fever is a potentially fatal disease of humans and domestic animals caused by Rift Valley fever virus (RVFV). Infection with RVFV in ruminants can cause near 100% abortion rates and recent outbreaks in naïve human populations have suggested case fatality rates of greater than thirty percent. To elucidate the roles that host proteins play during RVFV infection, proteomic analysis of RVFV virions was conducted using complementary analytical approaches, followed by functional validation studies of select identified host factors. Coupling the more traditional Gel LC/MS/MS approach (SDS PAGE followed by liquid chromatography tandem mass spectrometry) with an alternative technique that preserves protein complexes allowed the protein complement of these viral particles to be thoroughly examined. In addition to viral proteins present within the virions and virion-associated host proteins, multiple macromolecular complexes were identified. Bioinformatic analysis showed that host chaperones were among over-represented protein families associated with virions, and functional experiments using siRNA gene silencing and small molecule inhibitors identified several of these heat shock proteins, including heat shock protein 90 (HSP90), as important viral host factors. Further analysis indicated that HSP inhibition effects occur during the replication/transcription phase of the virus life cycle, leading to significant lowering of viral titers without compromising the functional capacity of released virions. Overall, these studies provide much needed further insight into interactions between RVFV and host cells, increasing our understanding of the infection process and suggesting novel strategies for anti-viral development. In particular, considering that several HSP90 inhibitors have been advancing through clinical trials for cancer treatment, these results also highlight the exciting potential of repurposing HSP90 inhibitors to treat RVF.

## Introduction

Rift Valley fever is a potentially fatal disease of humans and domestic animals caused by the Bunyavirus (genera Phlebovirus) Rift Valley fever virus (RVFV) [1]. RVFV causes near 100% abortion rates in pregnant ruminants and statistics from recent human outbreaks suggest that the case fatality rates have been dramatic (>30%) in naïve populations [1]. The virus is typically transmitted to vertebrate hosts from infected mosquito vectors, although infection may also result from contact with infected animal tissues [1]. RVFV is broadly distributed throughout the African continent, causing major epizootic episodes in southern, western and northern Africa [2]–[7]. Additionally, surveillance programs have also detected Rift Valley fever virus in the East African countries of Senegal, Mali and Guinea [1], and large outbreaks have also occurred in the Middle East and Asia. RVFV represents a major public health risk due to its broad distribution and potential lethality. Furthermore, due to its virulence and ease of aerosolization, RVFV is a potential bio-weapon and is categorized by the Centers for Disease Control and *National Institute of Allergy and Infectious Diseases* as a category A priority pathogen. To date, there are no approved therapies or vaccines to treat the disease in humans.

The RVFV genome consists of single-stranded, negative sense RNA organized into three differently sized segments: Large (L), Medium (M) and Small (S). The L segment encodes a single protein; a large multi-domain, RNA-dependent RNA polymerase. The M segment codes for two glycoproteins, termed G_N_ and G_C_ that make up the protein component of the viral envelope. Additionally, two major non-structural proteins, a 78 kDa protein (NSm1), and a14 kDa protein (NSm2), are transcribed from M utilizing alternative start codons. The S segment codes for the nucleocapsid protein N, which interacts with viral RNA and plays indispensible roles in RNA replication and transcription [8]. Interestingly, studies have established that the S segment of RVFV uses an ambisense strategy to code for an additional nonstructural protein (NSs) that accumulates in the host nucleus, polymerizes and forms filamentous structures [9], [10]. The NSs protein product does not get packaged within the RVFV virions and both the non-structural proteins NSs and NSm are dispensable for virus growth in tissue culture. Thus, the viral protein content of RVFV virions obtained from mammalian cells may be limited to the following; L, G_N_, G_C_, and N [1].

Virions contain all the molecular machinery needed to enter and exit host cells. Given the complexity of these processes and the simplicity of viral proteomes, it is likely that host proteins play major roles in infection. To date, proteomic analysis of Bunyavirus virions have not been reported; other studies conducted on a divergent group of viruses such as HIV, Epstein-Barr, vaccinia, murine cytomegalovirus, human cytomegalovirus, influenza, Ebola, Marburg and Kaposi’s sarcoma-associated herpes virus have consistently detected multiple host proteins [11]–[18]. For the most part, the roles that these proteins play in the biology of these viruses remain hazy, though in a few instances important functions have been established. For example, the peptidyl-prolylisomerase cyclophilin A interacts with the HIV Gag protein, and reduced virion-associated levels of this protein correlated with reduced viral infectivity [19], [20]. Additionally, the host SAP18-HDAC1 (Sin3a associated protein 18 kD- histone deacetylase 1) complex is recruited into HIV-1 virions and is functionally required for viral replication [21]. Therefore, proteins associated with virions can act as important or essential host factors for viral infection and represent novel targets for anti-viral therapeutic development.

In this study, we have used complementary approaches to characterize the proteome of RVFV virions of MP-12 strain [22] and have performed initial functional characterizations to demonstrate the importance of several identified host heat shock proteins for the process of infection. In one approach, we used Gel LC/MS/MS (SDS PAGE following by liquid chromatography tandem mass spectrometry), the most widely used approach to identify proteins in viral particles. In a complementary approach, we used an alternative strategy that allows isolation of protein complexes and has been traditionally used to investigate other biological systems, namely mitochondria [23]–[27]. Multiple host proteins were isolated and the use of Blue Native polyacrylamide gel electrophoresis (Blue Native PAGE) in the latter approach identified novel multi-protein complexes associated with RVFV virions. Bioinformatic analyses of the data suggested that many of the identified virion-associated proteins might play critical roles during different stages of RVFV infection, providing insight into poorly understood host-vial interactions. In particular, host chaperones were significantly enriched in purified RVFV preparations, and functional experiments using siRNA gene silencing and chemical inhibitors identified multiple chaperones that operate as important host factors during RVFV infection. Because the association of select HSPs with RVFV virions could be either indicative of a process important for integrity or function of released virions, or instead a process important for one or more stages of the virus life cycle during which it interacts with the host machinery (e.g., entry, amplification, budding), we performed experiments to address these two alternatives. The results presented here show a lack of adverse effects on the functional integrity or capacity of fully formed virions, and instead provide evidence that HSPs are important during the early phase of infection, corresponding to the post-entry replication/transcription phase of the virus life cycle.

## Materials and Methods

### Antibodies, Inhibitors, Cells and Virus

Antibodies specific for protein disulfide isomerase (ab5484), heat shock protein 90 (ab1429), heat shock protein 70 (ab47455), cytochrome c oxidase subunit VIb (ab54575), annexin A1 (ab33061), annexin A2 (ab41803), annexin A4 (ab33009), histone H4 (ab7311), peroxiredoxin 6 (ab16947), and CCT6 (ab140142) were obtained from Abcam (Cambridge, MA). Glucose-regulated protein 78 antibody (610978) was obtained from BD biosciences (San Jose, CA). Antibodies specific for HSP90β (5087) and CCT2 (3561) were obtained from Cell Signaling Technology (Beverley, MD). Antibody specific for HSPA8 (PA5-24624) was from Thermo Scientific Pierce products (Rockford, IL). The monoclonal antibody that recognizes the N-terminal glycoprotein (Gn) from Rift Valley fever virus was obtained from the USAMRIID hybridoma facility (Ft. Detrick, MD). 17-(Allylamino)-17-demethoxygeldanamycin (17-AAG) and 1,2-Bis(2-aminophenoxy) ethane-N, N, N′, N′-tetra acetic acid (acetoxymethyl ester) (BAPTA-AM) was obtained from Sigma (St Louis, MO). Heat Shock Protein Inhibitor I (KNK437) was obtained from EMD Biosciences/Calbiochem (Darmstadt, Germany). Vero cells were maintained in *Dulbecco’s modified* Eagles medium (Gibco, San Diego, CA) supplemented with 10% fetal bovine serum (HyClone, South Logan, UT). The MP-12 attenuated vaccine strain of RVFV was obtained from the Salk Institute, government services division (Swiftwater, PA).

### Purification of Rift Valley Fever Virus

Vero cells were grown to 80% confluency in ten 175 cm^2^ flasks and infected with RVFV virus at a multiplicity of infection (MOI) equal to 0.1. At 72 hours post-infection (p.i.), supernatants were harvested and cellular debris removed by centrifugation in a JLA 10.500 rotor (Beckman Coulter, Fullerton, CA) (4°C, 10 minutes, at 2600×g). Virus was precipitated from the clarified supernatant by adding polyethylene glycol (PEG) to a final concentration of 5% and stirring the solution overnight at 4°C. The precipitated virus was pelleted by centrifugation in the JLA 10.500 rotor (4°C, 30 minutes, at 2600×g), and the supernatants were decanted and viral pellets resuspended in a minimal (∼1 ml) volume of sterile PBS. Viral suspensions were applied to sterile 10–60% sucrose gradients and ultra-centrifuged for two hours at 32,000 rpm (126,000×g) in a SW-41 swinging bucket rotor (Beckman Coulter, Fullerton, CA). A distinct band corresponding to RVFV was removed and re-centrifuged overnight at 22,000 rpm in a JA 20 rotor (Beckman Coulter, Fullerton, CA) to pellet the virus. The supernatants were discarded and the RVFV was resuspended in a minimal volume of PBS, separated into aliquots and frozen at −80°C until analysis.

### LC MS/MS Protein Identification of Blue Native Complexes

Protein extracts prepared from solubilized RVFV virions were separated by Blue Native PAGE (see “Blue Native PAGE separation of native viral protein complexes” for details). Following staining using Coomassie blue, individual bands were excised from the gel, placed into individual tubes, and after destaining were proteolyzed overnight by trypsin at 37°C. Peptides were extracted using 70% acetonitrile/0.1% trifluoroacetic acid and desalted using C-18 Zip Tips (Millipore; Billerica, MA). Digestates were then lyophilized and resuspended in 0.1% trifluoroacetic acid before LC/MS/MS analysis. Peptide/protein identification was performed by nanoflow reverse phase LC/MS/MS using a nanoflow LC system (Agilent 1100; Agilent Technologies, Wilmington, DE) coupled online with a linear ion trap MS (LTQ; Thermo Electron; Waltham, MA). Micro-reversed phase LC columns were slurry packed in-house with 5 µm of 300-Å pore size C-18 phase (Jupiter, Phenomenex; Torrance, CA) in a 75-µm i.d. × 10-cm fused silica capillary with a flame pulled tip. After sample injection, the column was washed for 20 min with 98% mobile phase A (0.1% formic acid/water) at 0.5 µl/min, and peptides were eluted using a linear gradient of 2–42% mobile phase B (0.1% formic acid/acetonitrile) for 40 min at 0.25 µl/min and then 98% mobile phase B for 10 min. The linear ion trap MS was operated in a data-dependent mode in which each full MS scan was followed by MS/MS scans in which the five most abundant molecular ions were dynamically selected for collision-induced dissociation using normalized collision energy of 35%. Tandem mass spectra were searched against a combined database consisting of the mature proteins coded by RVFV and the UniProt human proteomic database from the European Bioinformatics Institute (http://www.ebi.ac.uk/integr8) using SEQUEST (Thermo Electron; Waltham, MA). Because of significant homology between primate and human proteins, the human protein database could be used to identify virion-associated host proteins from Vero cells, which are derived from African green monkey. Mass tolerance for the Blue Native PAGE LTQ analysis was MS value of 0.5 Da and MS/MS value of 0.8 Da. The fully tryptic peptides possessing SEQUEST cross correlation scores >2.0 (+1), 2.3 (+2), or 3.5 (+3) and ΔCn values >0.1 were considered legitimate identifications. These experiments were performed through a contract research agreement with the laboratory of proteomics and analytical technologies, advanced technologies program, SAIC-Frederick Inc. (Frederick, MD).

### Identification of Proteins by GEL LC/MS/MS

Purified virions were resuspended in SDS sample buffer (Invitrogen, Carlsbad, CA), denatured by boiling for 5 minutes and resolved by SDS PAGE on 10–20% gradient polyacrylamide gels (Invitrogen; Carlsbad, CA). Proteins were visualized by Imperial Coomassie blue staining (Thermo Scientific Pierce Protein Research; Rockford, IL) and gel sections were excised at positions along the length of the sample lane. Gel pieces were destained, dehydrated and shipped to NextGen Sciences (Ann Arbor, MI) for analysis. Samples were reductively alkylated by incubating samples in 2.5 mM DTT for 1 hour at 60°C and adding iodoacetamide to 10 mM for 1 hr incubation in the dark. Samples were in-gel digested with trypsin for 4 hours at 37°C and supernatant analyzed directly by LC/MS/MS on a Thermo Fisher (Waltham, MA) LTQ Orbitrap XL. Hydrolysate (30 µl) was loaded onto a 5 mm 75 µm ID C12 vented column (Jupiter Proteo, Phenomenex; Torrance, CA) at a flow-rate of 10 µL/min and eluted via a 30 minute linear gradient elution (0–50% acetonitrile, 0.1% formic acid) over a 15 cm 75 µm ID C12 column (Jupiter Proteo, Phenomenex) at 300 nL/min. The mass spectrometer was operated in data-dependent mode, selecting the six most abundant ions for MS/MS during each mass scan (60,000 FWHM resolution). Tandem MS data were searched using a local copy of Mascot (Matrix Science, London, UK) and used to generate deconvoluted data (DAT) files that were processed in the Scaffold algorithm (Proteome Software, Portland, OR). For the Gel LC/MS/MS Orbitrap analysis, the mass tolerance was MS value of 5 ppm and MS/MS value of 0.8 Da. Protein identifications that matched at least 2 peptides with minimum probabilities of 90% at the protein level and 50% at the corresponding peptide level were considered significant identifications. For protein identifications listed in Table S1 in File S1, the entire available database was searched in order to expand the search parameters and include the hits that have homologous sequences in other non-primate organisms.

We also performed a control experiment to catalogue the host proteins that may co-purify along with the virions following their release into the culture medium by dead or dying cells. Vero cells were either infected with RVFV or were subjected to multiple freeze thaw cycles in the absence of any infection in order to achieve cell lysis, as verified by microscopic examination of the cells after the last freeze thaw treatment. Culture supernatants were collected as described above and the RVFV virions and control sample were purified in parallel using the identical RVFV purification method described above (see “Purification of Rift Valley fever virus” for details). For the control sample, the same region of the sucrose density gradient was recovered to which RVFV virions migrate. The purified sample was run on a 4–12% Novex BIS-TRIS PAGE gel (Invitrogen), and the sample lane was excised and divided into 10 equally sized bands. Gel slices were cut into 2–3 mm cubes, washed 3X with 200 µl 50 mM ammonium bicarbonate, pH 8.0 and incubated in 100% acetonitrile for 15 minutes and dried in a speed-vac. Samples were subsequently reduced with 50 mM DTT at 56°C for 45 minutes and then alkylated with 55 mM iodoacetamide for 1 hour at room temperature. The material was again dried and rehydrated in 10 µL of a 12.5 ng/µl modified sequencing grade trypsin solution (Promega, Madison, WI). 75 µl of 50 mM ammonium bicarbonate, 10% acetonitrile, pH 8.0 was added and the samples were incubated overnight at 37°C. Peptides were extracted 2X with 50 µl 50% acetonitrile, 5% formic acid and dried in a speed-vac. Digests were resuspended in 20 µl Mobile phase A (0.1% Formic Acid, 0.005% heptafluorobutyric acid) and 10 µl was loaded onto a 15-cm×0.075 mm Easy-Spray PepMap column (Thermo Electron). Peptides were eluted over a period of 45 minutes, by applying a 5–65% linear gradient of Mobile phase B (Acetonitrile, 0.1% Formic Acid, 0.005% HFBA) at a flow rate of 300 nl/min. An LTQ OrbiTrap Elite (Thermo Electron, San Jose, CA) was run in a data-dependent collection mode with an instrument method composed of a single segment and 16 data-dependent scan events with a full MS scan followed by 15 MS/MS scans of the highest intensity ions. Normalized collision energy was set at 35, activation Q was 0.250 with minimum full scan signal intensity at 1×105 with no minimum MS2 intensity specified. Dynamic exclusion was turned on utilizing a three minute repeat count of 2 with the mass width set at 1.0 m/z. Protein searches were performed with MASCOT (Matrix Science, London GB) using a mixed taxonomy RVFV and Human subset database of the non-redundant protein database from National Center for Biotechnology Information (NCBI) web site (http://www.ncbi.nlm.nih.gov/). Search parameters included fixed carbamidomethylation of cysteines; trypsin digestion with two missed cleavages; variable modifications including oxidized methionines, STY phosphorylation, Gly-Gly (K). Precursor mass tolerance of 50 ppm and a product ion mass tolerance of 0.5 Da were used.

### Immunoblot Analysis

Immunoblot analysis was conducted on two sample types:1) Crude cell lysates; 2) RVFV virions. Crude cell lysate samples were made by growing Vero cells to high density in 6 well plates, collecting cells with a brief low speed centrifugation and resuspending cells in lysis buffer (50 mM Tris (pH 7.5), 280 mMNaCl, 0.2 mM EDTA, 2 mM EGTA, 1% Triton X-100, 1 mM dithiothreitoland protease inhibitors (Complete; Roche, Basel, Switzerland)). Cell resuspensions were frozen at −80°C overnight, thawed the next morning and centrifuged (4°C, 15 minutes, at 16,000×g) to remove cellular debris. Bradford assay (Biorad; Hercules, CA) was used to measure protein concentration, using bovine serum albumin as a standard. For experiments where Vero cell lysates were used as positive controls, 20 µg of protein was diluted in SDS sample loading buffer (Invitrogen; Carlsbad, CA), boiled for 5 minutes and analyzed. RVFV virions were prepared as described earlier. Prior to analysis, 1 µl of virus, corresponding to approximately 5 µg of total virion protein, was solubilized by adding 9 µl SDS sample loading buffer and boiling the sample for 5 minutes. Immunoblot analysis was conducted by resolving protein samples on 10–20% gradient polyacrylamide gels (Invitrogen; Carlsbad, CA) and transferring proteins to PVDF membranes. Individual proteins or classes of post-translational modifications were detected using primary antibodies in conjunction with appropriate species-specific horseradish peroxidase labeled secondary antibodies. Immunoblot images were visualized and digitized with the Storm gel imaging system (GE Healthcare, Waukesha, WI).

### Blue Native PAGE Separation of Native Viral Protein Complexes

Experimental protocols developed by Schagger and others to resolve electron transport complexes in isolated mitochondria were adapted to resolve native protein complexes in solubilized virions [23]–[25], [27]. Purified RVFV virions were solubilized by diluting virus (10 µl containing approximately 100 µg of protein) into solubilization buffer (50 mM NaCl, 50 mM imidazole, 5 mM 6-aminohexanoic acid and 1% triton X-100 or Igepal CA 630 (final concentrations) and allowing the suspension to incubate on ice for 20 minutes. Following incubation, a 10 minute centrifugation (16,000×g, 4°C) was used to pellet insoluble material. Supernatant (25 µl) was removed with a micropipette and mixed with 0.625 µl sample buffer (500 mM 6-aminohexanoic acid, 0.05%Coomassie G-250, and 50 mM Bis-Tris pH 7.0). The mixture was incubated at room temperature for 5 minutes prior to analysis. Samples were then loaded onto precast 4–12% polyacrylamide gels (Invitrogen, Carlsbad, CA) and subjected to electrophoresis. Initial electrophoresis conditions utilized a discontinuous buffer system consisting of “Deep Blue” cathode buffer (50 mM tricine, 15 mMBis-Tris pH 7.0 and 0.02% Coomassie G-250) and an anode buffer (50 mM Bis-Tris pH 7.0). Samples were then run at 100V until the dye front had migrated approximately 20% down the length of the gel. At this point, the “Deep Blue” cathode buffer was discarded and replaced with “Light Blue” cathode buffer (50 mMtricine, 15 mMBis-Tris pH 7.0 and 0.002% Coomassie G-250) and fresh anode buffer. Following buffer exchange, the gels were run at 200V until the dye front reached the bottom of the gel. The gel was then removed, washed with three water washes (5 minutes/wash) and re-stained with Imperial protein stain (Thermo Scientific Pierce Protein Research, Rockford, IL). Stained native gels were then photographed and native protein complexes were excised for MS analysis. For siRNA knockdown followed by BLUE Native PAGE analysis, siRNA treatments were performed as is described in the next section. Approximately 10^6^ cells per well of a 6 well plate were used for each condition and 1.5 ml of media from each well was used to obtain a viral pellet on a 0.5 ml 20% sucrose cushion.

### siRNA Gene Silencing of Host Chaperones

HeLa cells were transfected in 96-well plates (8,000 cells per well) with 80 nM small interfering RNA (siRNA) (Silencer Select, Applied Biosystems by Life Technologies; Carlsbad, CA) constructs targeting the gene coding regions of a select set of chaperones identified during proteomic experiments. Validated constructs, targeting glyceraldehyde 3-phosphate dehydrogenase (GAPDH), heat shock 70 kDa protein 8(HSPA8) and negative-control “scrambled” siRNA, were used in singlicate. The remaining genes were assayed using duplicate sets of siRNA constructs (i.e., two separate siRNA constructs tested against each target gene). Since these duplicate sets had not been validated by the manufacturer, we quantified the transcripts of their respective HSP target genes using quantitative reverse transcriptase PCR (qRT-PCR) in order to evaluate their knockdown capacity. We also assayed the pre-validated siRNA constructs to confirm the manufacturer’s validation. Forty eight hours after transfection of siRNAs, RNA was harvested using the Qiagen RNeasy mini RNA isolation kit per manufacturer’s instructions and purified RNA was measured using a NanoDrop 2000 Spectrophotometer (Thermo Scientific). qRT-PCR assays were performed under the following conditions; 20 µl reaction mix contained 0.4 µl ROX Reference dye, 1 µl of RNA UltraSense Enzyme Mix, 1 µl TaqMan primer/probe (Life Technologies), 3.6 µl nuclease-free water, 4 µl Reaction Buffer, and 10 µl (25 ng) of extracted RNA. The RT step involved incubation at 50°C for 15 min. The PCR conditions included an initial denaturation of 95°C for 2 min followed by 40 cycles of 95°C for 15 seconds and 60°C for 30 seconds. The target gene transcript levels were normalized against housekeeping GAPDH gene expression. Relative expression levels were determined using the comparative threshold cycle (*C_T_*) method. Each condition was tested in triplicates and the experiment was independently repeated. The results were averaged and numerical data was analyzed using student t-test (P<0.05). We also analyzed the effects of the HSP siRNA knockdowns at the protein level. For performing the knockdowns, the same procedure described above was followed except that HeLa cells were transfected in 24-well plates (32,000 cells per well). Forty eight hours after transfection, the cells were washed with PBS and total cell extracts were recovered and analyzed by Western blot analysis. Protein signal levels were determined using densitometry measurements and normalization for each lane to account for potential loading differences was achieved based on the densitometry values for GAPDH in the same lane.

### Analysis of the Effects of siRNA Gene Silencing of Host Chaperones during RVFV Infection

To screen for the knockdown effects of the siRNAs on the level of RVFV infection, both high content imaging assays of infected cells and qRT-PCR measurements of viral titers were performed. Both types of assays were independently repeated and the collected data for each condition was averaged. For high content imaging analysis, infection assays were conducted as described previously [28], [29]. Briefly, cells were transfected with individual siRNA constructs, incubated for 48 hrs, and infected with the MP-12 strain of RVFV. Following 24-hrs incubation, the percentage of infected cells and the percentage of viable cells were measured by high content immunofluorescence imaging protocols as described [28], [30]. Briefly, formalin-fixed infected cells were blocked with 3% BSA prepared in PBS for 1 hr and stained using the murine monoclonal antibody 4D4 that detects the Gn portion of the glycoprotein of RVFV. After washing with PBS, the cells were stained for 1 hr at RT with Alexa 488-conjugated goat anti-mouse secondary antibody (1∶1,000; Life Technologies) to visualize primary antibody. Cell nuclei and cytoplasm were labeled with Hoechst 33342 (Life Technologies) and HCS CellMask Red or Deep Red (Life Technologies) at a 1∶10,000 dilution. High-content quantitative imaging data were acquired and analyzed on an Opera confocal reader model 3842 (Quadruple Excitation High Sensitivity {QEHS}; PerkinElmer) at two exposures using X 10 air objective or X 40 water objective. Analyses of the images were accomplished within the Opera or Columbus environment using standard Acapella scripts. For each condition, images from 6 fields/well (∼1700 cells) were acquired with the script calculating the percent positive cells and the mean fluorescence intensities in the cytoplasmic, membrane, or nuclear region. To ensure that the siRNA treatments do not result in cytotoxicity, percent reduction in cell number was quantified as part of the imaging analysis, and the numerical data was analyzed using student t-test.

The effects of siRNA treatments on RVFV titers were also determined independently by qRT-PCR using TaqMan-based probe sequences as previously described [31]. Each condition was tested in triplicates and the experiment was independently repeated. The results were averaged and numerical data was analyzed using student t-test (P<0.05).

### Evaluation of Select Chaperone Inhibitory Compounds in RVFV Infection Assays

Vero cells were pretreated for 2 hours with equivalent volumes of either DMSO (control), 17-AAG (10 µM), BAPTA (10 µM), or KNK437 (10 µM), prior to infection with the MP-12 strain of RVFV at an MOI of 0.1. Following infection, cells were washed with PBS and media containing either DMSO or compound was added back to the cells. Twenty-four hours later, supernatants were collected for analysis of viral RNA. Viral RNA was extracted using Ambion’s (Applied Biosystems/Ambion, Austin, TX) MagMAX viral RNA extraction kit and quantitated using q-RT-PCR with primers and probe for G2, originally described by Drosten *et al* [31]. Q-RT-PCR assays were performed using Invitrogen’s (Carlsbad, CA) RNA UltraSense One-Step Quantitative RT-PCR System on an ABI 7000 sequence detection system. Absolute quantitation of RVFV RNA was performed using a standard curve where the RNA concentration is expressed in genomic copies/reaction. Currently, our assay detects 10^2^ copies per reaction 100% of the time and 10^1^ copies 50% of the time, which is in agreement with other published methods of PCR detection [32]. Cell viability assays were performed using CellTiter-Glo Luminescent Cell Viability Assay (Promega) according to the manufacturer’s instructions. DTX 880 multimode detector (Beckman Coulter) was used for detection of Luminescence.

### Time of Addition Studies and Plaque Assays

Vero cells were treated with HSP inhibitors 17-AAG or BAPTA-AM, or with vehicle only (DMSO), at various times post MP-12 infection (2 hr, 4 hr, 8 hr, and 14 hr p.i.). The inhibitors were maintained for the duration of the experiment. Infection was with MP-12 (MOI 0.1) for 1 hr at 37°C, after which each inoculum was removed and replaced with fresh medium (either with or without inhibitors). Viral RNA was extracted from 100 µl of supernatant from each sample and measured by qRT-PCR analysis, as described above. Functional RVFV yields for supernatants from DMSO-treated control sample and HSP-inhibited samples with the most significant decrease in viral load (4 hours p.i.) were also determined by a standard plaque assay, as previously described [30].

### Luciferase Assays

HepG2 cells were either left untreated or were pretreated before infection with vehicle only (DMSO), or 17-AAG, or BAPTA-AM, or a combination of 17-AAG and BATPA-AM. The cells were then infected with either RVFVrMP12-rLucmutant strain (NSs gene replaced by a Renilla Luciferase-encoding gene) [33], or regular MP-12 (negative control), at MOI 0.1. Mock conditions (no infection controls) with either no treatment or treatment with DMSO were also included. Following incubation for 1 hour at 37°C, the infectious culture media were removed, cells washed with PBS, and fresh medium was added back (either with or without the inhibitors). At 4 hours p.i., and 8 hours p.i., the media were removed from the wells, the cells were washed with Phosphate Buffer Saline (PBS), and Renilla Luciferase Assay lysis buffer (Promega) was used to lyse the cells for measuring luminescence levels. The Renilla-Glo Luciferase Assay System (Promega) was used following the manufacturer’s instructions. DTX 880 multimode detector (Beckman Coulter) was used for luminescence detection. Each condition was tested in triplicates and the experiment was independently repeated. The results were averaged and numerical data was analyzed using student t-test (P<0.05).

### Analysis of Functional Integrity of RVFV Virions Released from HSP-inhibited Infected Cells

The same batch of Vero cells was used for DMSO treated (control) and 17-AAG treated conditions. Identical number of cells was used for both conditions. Prior to MP-12 infection, Vero cells were either pretreated with 17-AAG or with a matching volume of DMSO, and were subsequently infected with MP-12 at MOI 0.1. Following infection, cells were washed with PBS and media containing either 17-AAG or DMSO was added back to the cells. Twenty-four hours later, supernatants were collected for measuring viral titers by both qRT-PCR and plaque assays, following the procedure described above. The viral titer values obtained from plaque assays were then normalized to the amount of released virus measured by qRT-PCR analysis. Each condition was tested in triplicates and the experiment was independently repeated. The results were averaged and numerical data was analyzed using student t-test (P<0.05).

### Bioinformatic Analysis

Complexes identified from Native PAGE experiments (Tables S2–S4 in File S1) were annotated by searching databases of known protein-protein interactions (Ingenuity knowledge base, Ingenuity Systems, Redwood City, CA) and STRING V 8.0 (http://string.embl.de/)) and mapping these physical interactions into protein networks [34]. Lists of proteins corresponding to native complexes I–IV were submitted to each database individually. STRING queries only considered high confidence interactions (minimum score 0.700) based on experimental evidence (search parameters: experiments, databases and text mining). The Ingenuity knowledge base was searched by submitting lists to the search engine and only considering “direct” interactions between proteins. Search results between the two divergent databases were manually concatenated for creating networks of physical interactions.

### Network Generation, Canonical Pathway Enrichment

Entrez Gene symbols for all identified proteins were uploaded to Ingenuity Pathways Analysis (Ingenuity Systems, www.ingenuity.com). Against a reference set of all Ingenuity molecules, virion-associated proteins were assayed for enrichment of canonical pathways present in the Ingenuity Knowledge Base using Fisher’s exact test. Networks up to 70 molecules in size were algorithmically generated based on their connectivity within the Ingenuity Knowledge Base (IPA Network Generation Algorithm, White paper), including both direct and indirect interactions, and the highest scoring network was displayed.

## Results

### Purification of RVFV Virions

**Figure 1:**
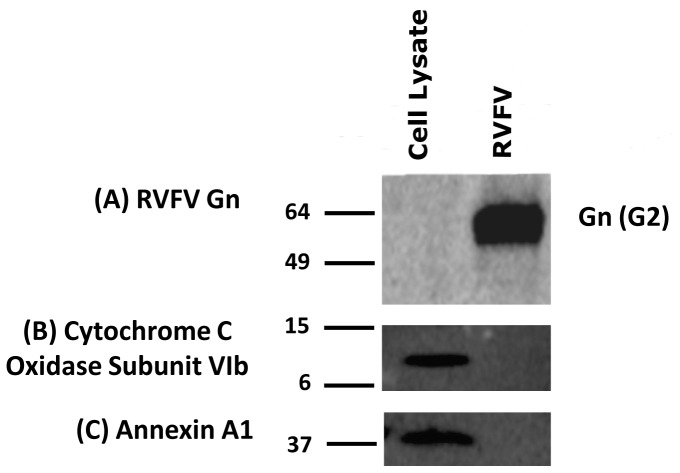
Evaluating the purity of viral preparations by Western blot analysis. Vero whole cell lysate (positive control) along with detergent solubilized RVFV virions were resolved by SDS PAGE, transferred to PVDF membranes and analyzed by Western blot. (A) Blots probed with antibodies against the RVFV glycoprotein Gn (G2) demonstrate that this protein is only present in solubilized virus. (B) The mitochondrial protein Cytochrome C Oxidase Subunit VIb is detected in whole cell lysate but not in purified virions. (C) The plasma membrane protein Annexin A1 is detected in whole cells but not in the viral preparation.

Identification of virus-associated host proteins requires purified virus. Therefore, a purification protocol involving PEG precipitation and sucrose density centrifugation was used to produce purified RVFV virions. To assess the purity of the viral preparations, virus and whole cell lysates made from uninfected host (Vero) cells were examined for select marker proteins (Figure 1). Immunoblot analysis of both sample types detects the RVFV glycoprotein Gn within purified virions but not in uninfected control cells, confirming that preparations contain RVFV. The purity of the virions was examined using immunoblot analysis against the mitochondria specific protein Cytochrome C Oxidase Subunit VIb and the plasma membrane marker annexin A1. Both proteins are present in whole cell lysates but are not detected in purified virions by Western analysis. The marker protein annexin A1 is one of the most abundant proteins in the plasma membrane and has been reported to be present in other virions and in membrane microvesicles [11], [35], [36]. Because of their small size, impurities in viral preparations are likely to be caused by plasma membrane microvesicles. However, the lack of annexin A1 Western signal in RVFV preparations suggests that this is not a significant concern in our case. Collectively, these data strongly suggest that the purified virions are largely free of whole cell or mitochondrial contamination, demonstrating that the RVFV preparation is purified and suitable for proteomic analysis.

### Characterization of Protein Content of RVFV Virions by GEL LC/MS/MS Analysis

**Figure 2:**
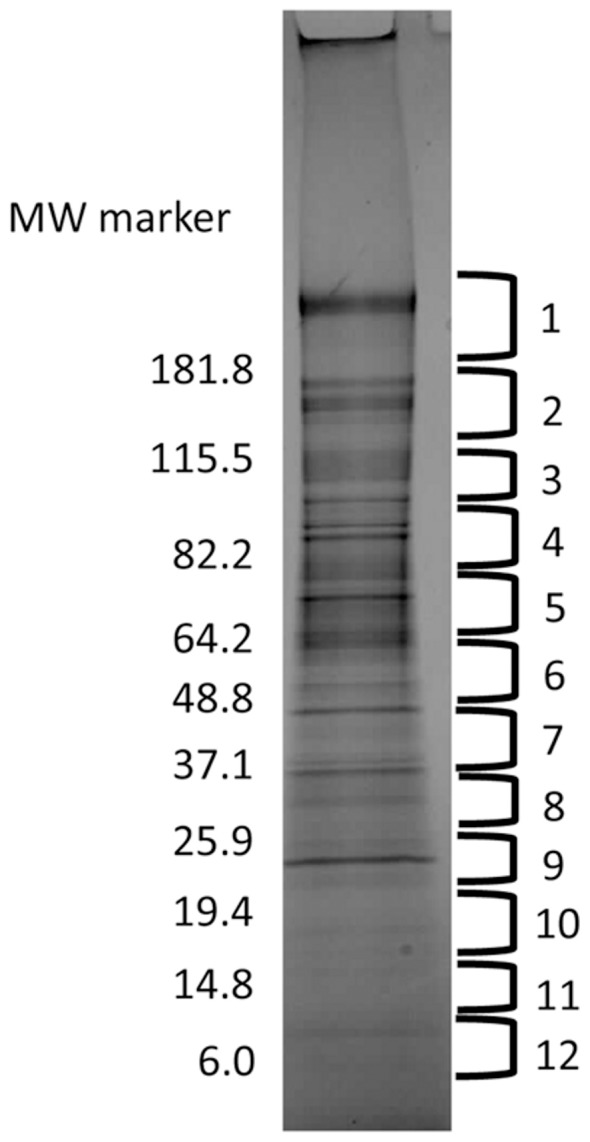
SDS PAGE separation of proteins in solubilized RVFV virions. Purified virions were denatured by boiling in SDS sample buffer (50 mM Tris-HCl (pH 6.8), 10% glycerol, 2% SDS and 0.0025% bromophenol blue) and resolved on a 4–20% acrylamide gel. The gel was then stained with Coomassie blue and protein bands excised at the 12 locations indicated on the figure. The molecular weights of protein standards are noted in the figure.

RVFV virions were analyzed by SDS PAGE-liquid chromatography tandem mass spectrometry (GEL LC/MS/MS). Viral particles were denatured by boiling in SDS sample buffer and resolved by electrophoresis through a polyacrylamide gradient (4–20%) gel. Protein bands were visualized by Coomassie Blue staining and gel segments excised as indicated (Figure 2). The protein content of each gel segment was analyzed by LC/MS/MS. It should be pointed out that MALDI analysis of the virion samples was also performed independently and indicated a high degree of overlap with the LC/MS/MS data set (data not shown). We also performed a control experiment in which total cell lysate from uninfected cells were purified in parallel with RVFV virions using the identical purification procedure described above (see “Purification of Rift Valley fever virions”). We had the opportunity to use a current state of the art MS instrument (LTQ OrbiTrap Elite) for this comparative study, allowing us to achieve even greater MS sensitivity. For the control sample, the same sucrose density gradient region to which RVFV virions migrate were recovered. The MS protein identifications fort the control sample were compared with the MS identifications for virion-associated host proteins. A total of 28 proteins were found to be common between the two data sets (Table S5 File S1). This list does not include any of the HSP proteins that are reported here or any of the other host proteins that are discussed in terms of potential relevance to RVFV infection, further indicating that their association with RVFV virions is not simply a co-purification artifact. It is also worth noting that this control experiment likely represents a high stringency condition in which higher levels of non-specific cellular proteins are recovered from the culture supernatant than are present during the infection condition. While there is a high level of cytopathic effects (CPE) at the time of culture supernatant harvest in late infection, this does not likely equate to a full release of the cellular contents into the media, as was achieved for the control sample through freeze-thaw cycles. For this reason, even for proteins listed in the Table S5 in File S1, very firm conclusions about lack of virion association may need to await further analytical experiments and characterizations.

Table S1 in File S1 and Table 1 list the identified host and endogenously encoded RVFV proteins respectively. For the identified proteins, the known molecular functions are also indicated in the tables. Viral proteins reported to be present in the virions were also detected by our MS analysis (Table 1).

**Table 1:** Endogenous viral proteins identified in RVFV virions.

Fraction Number (MWT range in kDa for excised gel slices)	Protein Name	Total Number of Peptides Detected with High Confidence
1 (190–230)	L Polymerase	4
1 (190–230)	Glycoprotein^1^	3
2 (120–190)	Glycoprotein	8
3 (90–120)	Glycoprotein	6
4 (70–90)	Glycoprotein	7
5 (55–70)	Glycoprotein	33
6 (40–55)	Glycoprotein	10
7 (30–40)	Glycoprotein	5
8 (27–30)	Nucleocapsid	5
8 (27–30)	Glycoprotein	2
9 (20–27)	Nucleocapsid	27
10 (15–20)	Nucleocapsid	7
10 (15–20)	Glycoprotein	3
11 (12–15)	Nucleocapsid	7
11 (12–15)	Glycoprotein	4
12 (6–12)	Glycoprotein	2

1 The mature N-terminal (Gn) and C-terminal (Gc) Glycoproteins could not be distinguished by this analysis as peptides from both regions were detected. These proteins are referred to collectively as “Glycoprotein”.

### Blue Native PAGE/LC/MS/MS Characterization of Native Protein Complexes in RVFV Virions

**Figure 3:**
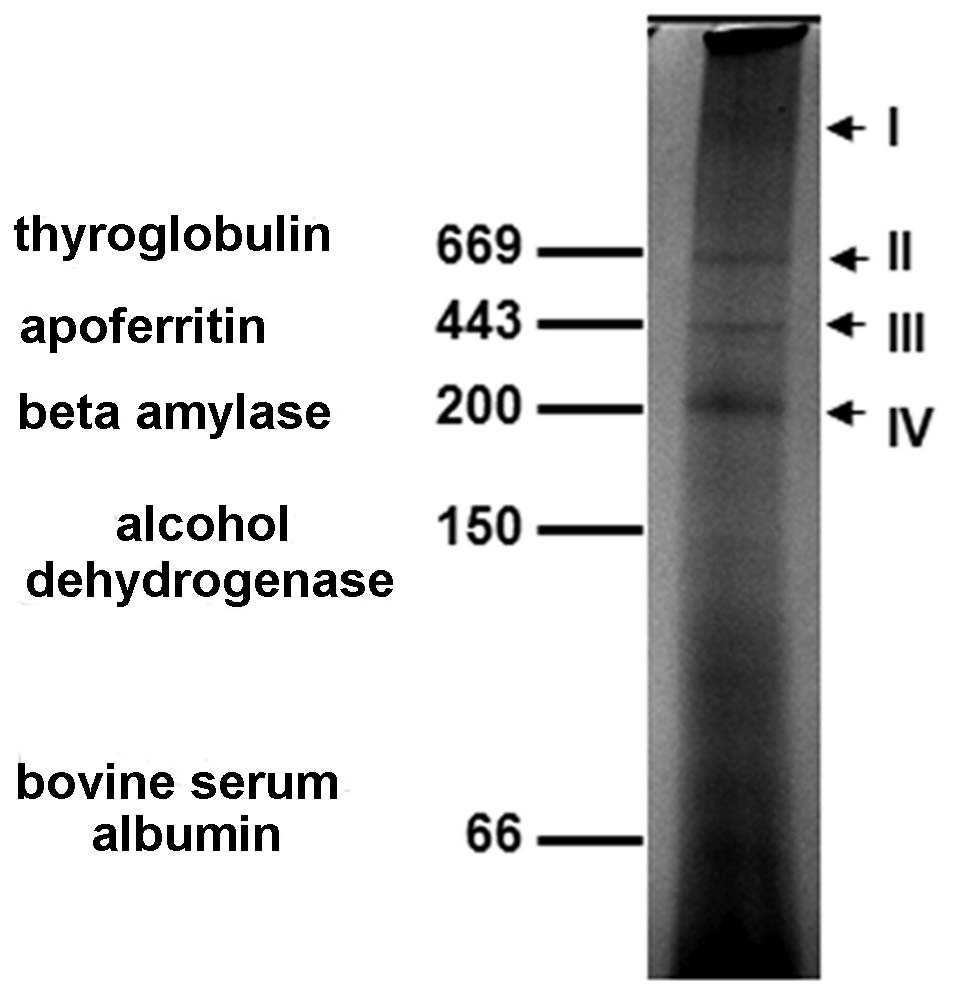
Blue native PAGE separation of high molecular weight protein complexes in solubilized RVFV virions. Purified virions were solubilized under native conditions by diluting virus into a buffer containing 1% Trition X-100 and removing insoluble material by centrifugation. Sample buffer containing Coomassie Blue G-250 was then added to supernatants and samples were subjected to Blue native polyacramide gel electrophoresis on 4–12% gradient gels. Native protein standards (thyroglobulin 669 kDa, apoferritin 443 kDa, beta amylase 200 kDa, alcohol dehydrogenase 150 kDa, and bovine serum albumin 66 kDa) were resolved in adjacent lanes. The position of the fastest migrating band for each protein standard is indicated on the figure. Four high molecular weight protein species were observed in solubilized virions; they are labeled I–IV on the figure.

Blue native PAGE separates individual proteins and protein complexes under non-denaturing conditions. This methodology was applied to RVFV virions by solubilizing virus in 1% Triton X-100 and resolving samples in the presence of buffers containing Coomassie blue G-250 (Figure 3). The results show four high molecular weight species (labeled I–IV) and several other lower molecular weight proteins resolved on a 4–12% acrylamide gel. RVFV virions solubilized with 1% IGEPAL CA 630 and resolved by Blue native PAGE yielded the same four high molecular weight complexes (data not shown). The proteins present within these complexes were identified using LC/MS/MS. Database searches identified multiple host proteins and the viral nucleocapsid protein (N) in complexes I, II and IV. In addition to the RVFV nucleocapsid protein, 23, 45 and 32 host proteins were respectively detected in complexes I, II and IV. Only a single protein (caspase 2) was identified in complex III. Tables S2–S4 in File S1 summarize the identification of proteins in these species.

### Immunoblot Identification of Host Proteins Associated with RVFV Virions

**Figure 4:**
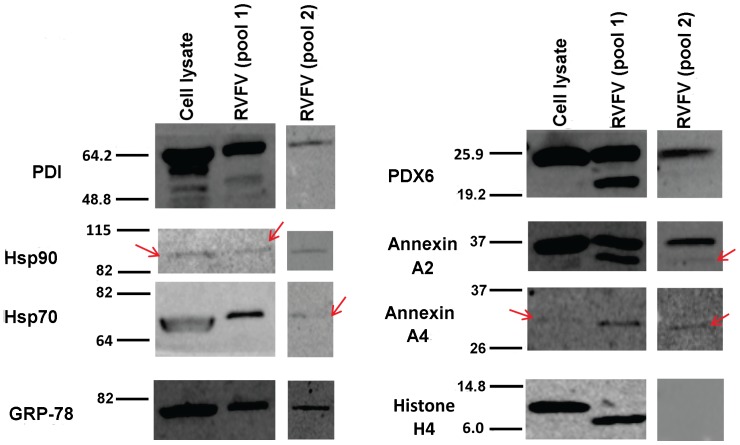
Detection of MS-identified host proteins in RVFV virions by Western blot analysis. Vero whole cell lysate (positive control) along with two different preparations of detergent solubilized virions were resolved by SDS PAGE and transferred to PVDF membranes for Western blot analysis using antibodies against eight cellular proteins. For the whole cell lysate, the total amount of protein applied to each well was 20 micrograms and for the virion preparations it was about 5 micrograms. All eight proteins were detected in positive control samples. Seven proteins were detected in both preparations of RVFV virions: protein disulfide isomerase (PDI), heat shock protein 90 (HSP90), heat shock protein 70 (HSP70), glucose-regulated protein 78 (GRP-78), peroxiredoxin 6 (PDX 6), annexin A2, and annexin A4. Histone H4 was only detected in one virion prep. Faint bands are demarcated with red arrow. For the HSP70 Western, it should be noted that because the amount of the HSP70 protein loaded in the cytoplasmic lysate lane is much greater than the virion lanes, the protein band mobility in this lane appears slightly different compared to the virion lanes.

Immunoblot analysis was used to confirm the presence of several well-characterized proteins identified by proteomic experiments. These proteins were also chosen because of the availability of well-characterized specific antibodies to probe them. Purified RVFV virions prepared from two independent preparations and control whole cell lysates from uninfected Vero cells were solubilized in SDS sample buffer, resolved by SDS-PAGE, transferred to PVDF membranes and probed with antibodies directed at various host proteins (Figure 4). Immunoblot experiments confirmed the presence of all eight host proteins in RVFV preparation 1, and seven of eight proteins from the second RVFV preparation. The concordance of these results suggests that the MS identifications, which are based on peptide analysis, accurately reflect the presence of the corresponding proteins in the purified virion preparations.

### Bioinformatic Analysis of Proteomic Data

**Figure 5:**
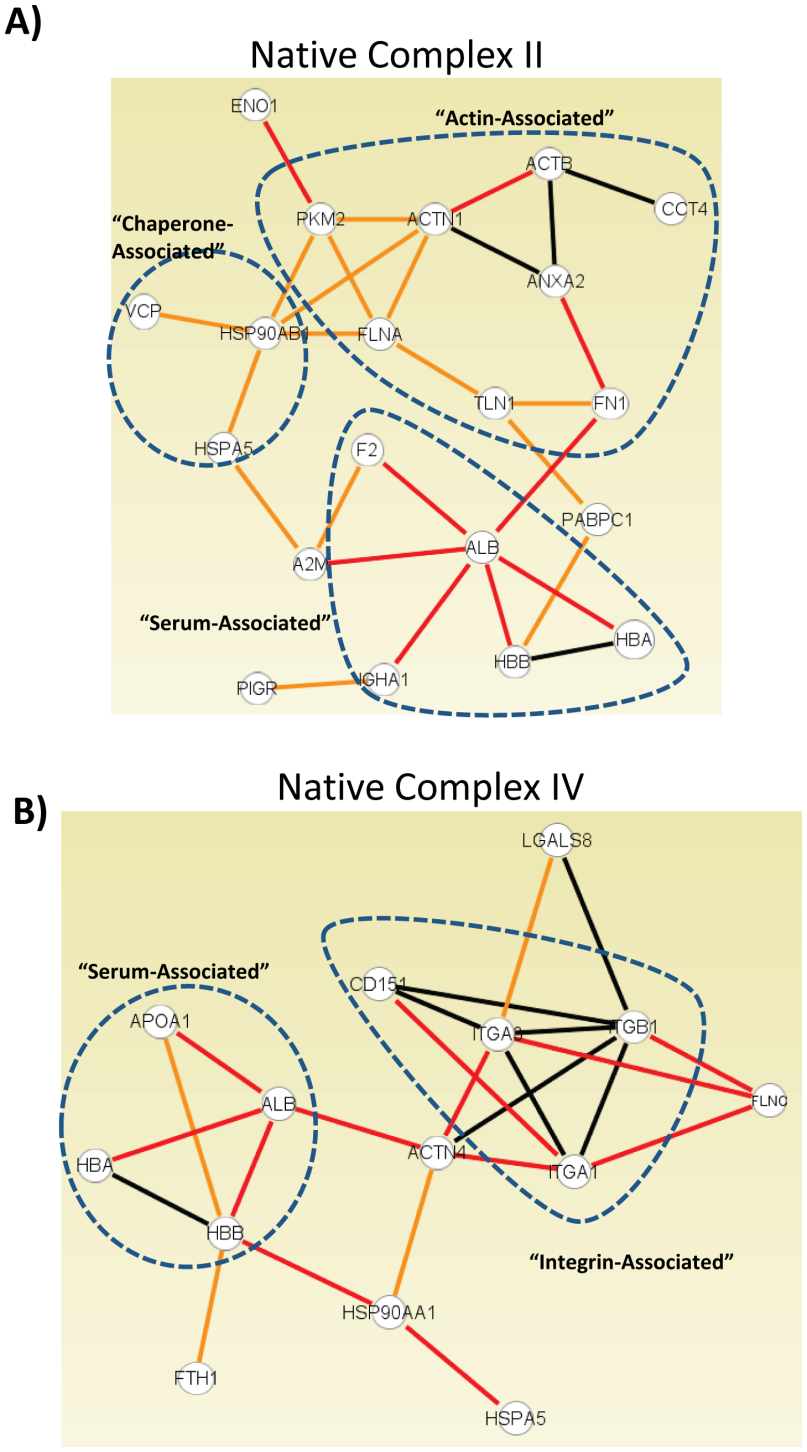
Protein-Protein interaction networks in RVFV complexes II and IV. Proteins in native complexes II and IV are involved in multiple interactions with other proteins in their respective complexes. Interactions were mapped using the Ingenuity knowledge base (orange edges), and the STRING database (red edges). Black edges represent interactions annotated by both sources. (A) Native complex II: ENO1 α-enolase, PKM2 pyruvate kinase muscle, ACTN1 actinin-α1, ACTB beta actin, CCT4 chaperone containing TCP1, IGHA1 Immunoglobulin heavy chain α, ANXA2 annexin A2, TLN1 talin 1, VCP valosin-containing (TER ATPase), HBB hemoglobin beta, HSP90AB1 HSP 90 α class B mem 1, FLNA filamin A, FN1 fibronectin1, PIGR polymeric immunoglobulin receptor, HSPA5 HSP 70, protein 5 (GRP-78), HBA hemoglobin alpha, F2 coagulation factor II, PABPC1 polyA binding protein, A2M α-2-macroglobulin, ALB albumin. (B) Native complex IV: LGALS8 galectin-8, CD151 CD151, ITGA3 Integrin α 3, ITGB1 Integrin β 1, ITGA1 Integrin α 1, HBB hemoglobin beta, ACTN4 actinin-α4, HBA hemoglobin alpha, HSP90AA1 HSP 90 α class A mem 1, FLNC filamin C, HSPA5 HSP 70, protein 5 (GRP-78), ALB albumin, APOA1 apolipoprotein A1, FTH1 ferritin polypeptide 1.

To analyze the validity of complexes identified by Blue native PAGE (Figure 3) and to gain insight into the functions of these RVFV-associated species, protein data sets corresponding to native species I, II and IV were compared against databases of known protein-protein interactions (Ingenuity Systems and STRING v 8.0). Complexes II and IV could be mapped to extensive continuous networks consisting respectively of 20 and 13 node physical interaction networks (Figure 5). Interactions that were determined using the STRING database are connected with red edges in the figure whereas those identified with Ingenuity are connected by orange edges, and those interactions that were common between both databases are shown as black connections. Thus, for these two complexes, nearly 40% of identified subunits (20/45 for complex II and 13/32 for complex IV) can be mapped based on previously reported data. Within each of these complexes there are sub-complexes that form extensive contacts and share biological functions. Complex II contains an “Actin-Associated” sub-complex made of 8 nodes that are involved in actin binding or interactions with other cytoskeletal elements. There is also a 3-node sub-complex that contains proteins with protein folding activity labeled as “Chaperone-Associated” and lastly a 5-node “Serum-Associated” sub-complex. Complex IV is composed of a 4-node “Serum-Associated” sub-complex and a 4-node “Integrin-Associated” sub-complex that contains three Integrin subunits and the Integrin associated protein CD 151.

**Figure 6:**
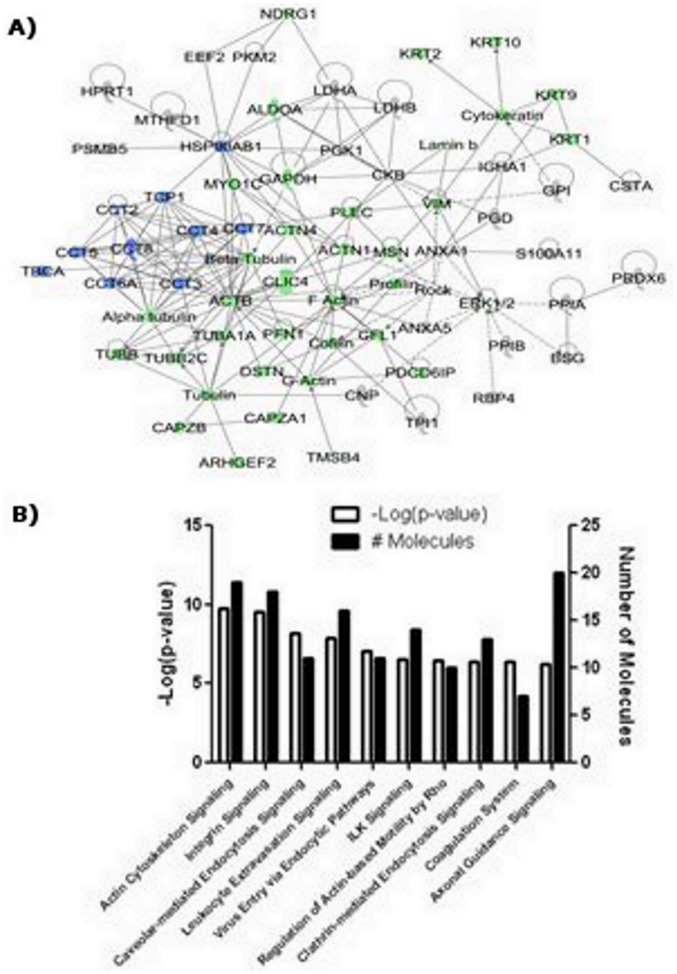
Ingenuity Analysis of all proteins identified in the study. (A) The complete data set was used to create the 69 node (largest discovered by our analysis) interaction network depicted in the figure. The nodes colored in blue designate chaperones and those in green designate cytoskeletal components. Solid lines represent proteins with known direct interactions, while indirect interactions are represented with broken lines. (B) The bar graph depicts the ten most enriched canonical pathways as a function of the enrichment score for the pathway (−Log (p-value)) and the number of molecules (proteins) represented within the pathway.

To gain insight into the functions that RVFV-associated host proteins play, the complete list of proteins identified by all experimental methods (Figures 2–3) was analyzed by a bioinformatic approach using Ingenuity Systems pathway analysis (IPA). First, interaction analysis was used to assess the connectivity of host proteins associated with RVFV virions. All non-redundant proteins identified were submitted to IPA and all known direct and indirect interactions were used to generate protein networks. The highest-scoring network contained 69 proteins (Figure 6A); the network contains many host chaperones and cytoskeletal components. A second approach searched over-represented canonical pathways (the co-occurrence of proteins within our data with a frequency much greater than that expected by chance). The top 10 over-represented canonical pathways are presented (Figure 6B) and graphed via the statistical parameter −Log (p-value) and the number of proteins from our data set represented in the pathway. The identified pathways are dominated by signaling pathways (integrin, cytoskeleton) and known pathways of viral entry (virus entry via endocytic pathway and caveolar/clathrin endocytosis signaling).

### Analysis of Chaperone Activity in RVFV Infection by Gene Silencing and Chemical Inhibitor Screening

**Figure 7:**
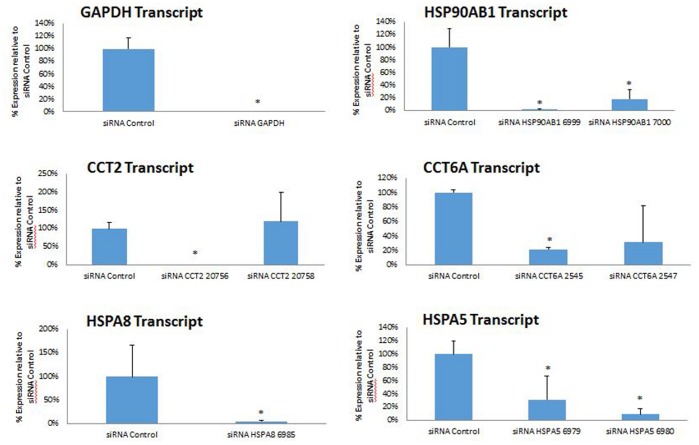
siRNA silencing of target host chaperones. Knockdown efficiencies of Silencer Select siRNA library (Life Technologies) were quantified, using qRT-PCR to measure the transcript levels of the respective target genes. HeLa cells were transfected with siRNA molecules targeting chaperone proteins, or negative control targets (GAPDH, scrambled siRNA), and RNA samples were purified 48 hours post transfection for analysis by qRT-PCR. Analysis was performed for both pre-validated single siRNA constructs and the constructs in the duplicate sets that represent independent siRNAs targeting the same gene and had not been previously validated. siRNA constructs that showed significant down-regulation compared to siRNA control are designated by asterisk (P<0.05). Data are average values from three independent assays.

**Figure 8:**
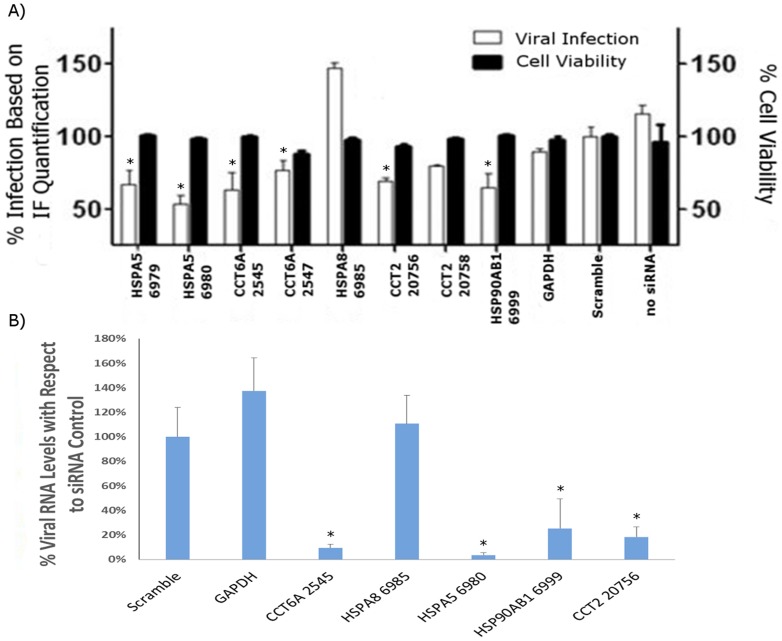
Effects of siRNA silencing of host chaperones on RVFV levels. (A) Silencer Select siRNA library (Life Technologies) was used to serially assess the importance of identified chaperones in RVFV infection. HeLa cells were transfected with siRNA molecules targeting chaperone proteins, negative control targets (GAPDH, scrambled siRNA) or “no siRNA” controls. Following 48 hr incubation, cells were infected with the MP-12 strain of RVFV and the extent of viral infection assessed by high content imaging, as described in the Methods section. The down-regulation of chaperone proteins indicated in the figure resulted in statistically significant effect on viral infection when compared to the scrambled siRNA, as indicated by the asterisk symbols (P<0.05). Negative controls corresponding to GAPDH, scrambled siRNA and no siRNA control did not impact RVFV infection. The experiment was repeated in three independent trials with samples assayed in duplicate. The displayed data is a representative of the entire sample set. (B) The effects of siRNA treatments on RVFV titers were also measured using qRT-PCR analysis. For each target HSP with a duplicate siRNA set, the siRNA construct with the highest knockdown efficiency was analyzed. HeLa cells were transfected with the siRNA constructs and after 48 hours the transfected cells were infected with the MP-12 strain. The culture supernatants were analyzed 24 hours after infection. Asterisks designate significant changes in viral levels (P<0.05).

**Figure 9:**
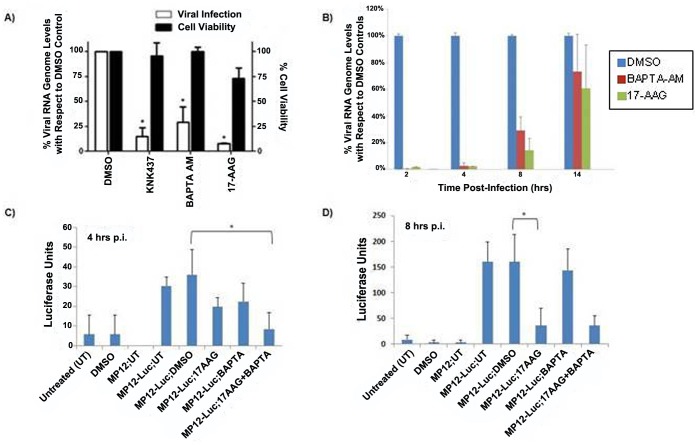
Effects of host chaperone inhibitors on RVFV levels and timing of HSP effects. (A) Vero cells were pretreated for 2 hours with equivalent volumes of either DMSO (control), 17-AGG (10 µM), BAPTA (10 µM), or KNK437 (10 µM), prior to infection with the MP-12 strain of RVFV (MOI 0.1). Following infection, cells were washed with PBS and media containing either DMSO or each of the compounds was added back to the cells. Twenty-four hours later, supernatants were collected and viral RNA was measured by qRT-PCR as described in the Methods section. Percent viral RNA is expressed as the percent of the DMSO control. The viability of the cells in the presence of 10 µM inhibitor or in DMSO (vehicle) was assessed by MTT assay, as described in the Methods section. Data are average values from three independent assays. (B) Time of addition studies were performed using 17-AAG and BAPTA-AM inhibitors to analyze the timing of HSP effects during RVFV infection. Vero cells were treated with HSP inhibitors, or with vehicle only (DMSO), at the indicated time points relative to MP-12 infection (MOI 0.1). Following infection, cells were washed with PBS and media containing either DMSO or each of the compounds was added back to the cells. qRT-PCR was performed to measure viral RNA levels in the supernatants collected 24 hours post-infection. Data are average values from three independent assays. (C), and (D) Viral inhibition following HSP inhibitor treatment was measured in real time. One hour prior to infection with rMP12-rLuc (MOI 0.1), HepG2 cells were treated with HSP inhibitors 17-AAG, or BAPTA-AM, or a combination of both inhibitors. Controls included no inhibitor treatment and no infection [Untreated (UT)], no inhibitor treatment but with infection using either the regular MP-12 strain that has no luciferase expression (MP12; UT) or with the luciferase expressing rMP12-rluc strain (Mp12-Luc; UT), and treatment with vehicle only, either without any infection (DMSO) or with infection using the rMP12-rluc strain (Mp12-Luc; DMSO). All samples were analyzed either at 4 hours p.i. (C) or at 8 hours p.i. (D), using the Renilla Luciferase assay. Data are average values from three independent assays. Asterisks designate significant changes in luminescence levels (P<0.05).

Chaperones were among the most over-represented proteins within our data set and have been previously shown to function as important viral host factors for other viruses [18], [37]. To assess the roles that the identified chaperones play in RVFV infection, a library of siRNA molecules targeting the chaperones identified from MS analysis was screened in an infection assay for effects on RVFV. Only the HSPs that showed an effect in the initial screen were analyzed further and are discussed here. For all the siRNA constructs, we measured the level of silencing of respective target HSP genes by qRT-PCR; both singlet siRNAs that had been pre-validated by the manufacturer, and duplicate siRNA sets consisting of two independent siRNAs targeting the same HSP, were analyzed. The results demonstrate that all the pre-validated siRNAs and at least one or both siRNAs in the duplicate sets give significant knockdown effects of their respective target genes (Figure 7). Furthermore, we analyzed the effects of siRNA knockdown on target HSPs at the protein level. Based on the results presented (Figure 7), the siRNA constructs with highest knockdown efficiency were used for this experiment. Validating the qRT-PCR data, the Western analysis results also show significant reduction in the levels of target HSPs, with a lowering of the protein levels in the approximate range of 62–80% depending on the specific HSP (Figure S1). For the initial screening of the effects of all the siRNAs on RVFV infection, we took advantage of the speed afforded by the high content imaging technique that has been used to measure infection level for RVFV and several other viruses [28], [30]. The results of this screen are presented (Figure 8A). Constructs that down-regulated HSPA5 (heat shock 70 kDa protein 5), HSP90AB1 (heat shock protein 90 kDa alpha (cytosolic), class B member 1), CCT6a and CCT2 (two CCTT-complex subunits) resulted in decreased RVFV infection, as judged by immunofluorescence/high content imaging analysis. For these constructs, the observed percent changes in immunofluorescence signal compared to the scrambled siRNA control were as follows: −32.54% and −46.40 percent for HSPA5 6979 and 6980 respectively; −36.95% and −23.225 percent for CCT6a 2545 and 2547 respectively; −30.73% and −19.94% percent for CCT2 20756 and 20758 respectively; and, −34.84% for HSP90AB1. Except for the CCT2 20758 construct, all were statistically significant changes (P<0.05). The effect on viral replication by the duplicate siRNA sets (i.e., two independent siRNA constructs targeting the same HSP gene) correlates well with their respective silencing efficiencies (Figures 7 and 8A); thus, in the duplicate sets, those siRNA constructs that give greater knockdowns of their target HSP also give a larger reduction in the level of infection, further indicating the specificity of the effects of the siRNAs on viral load. Interestingly, down regulation of HSPA8 (heat shock 70 kDa protein 8) showed the opposite effect and resulted in enhanced viral infection, with a 47.28% increase compared to the negative control scrambled siRNA. Negative siRNA controls (GAPDH and scrambled construct) had no significant impact on infection and are included for comparison purposes. Furthermore, statistical analysis of cell viability measurements indicated that the siRNA knockdowns did not cause cytotoxicity (P<0.05), (Figure 8A). We also used qRT-PCR to quantify siRNA silencing effects on viral levels and further confirm the high content imaging screen results. For this experiment, from each duplicate siRNA set, we selected the one that showed greater knockdown efficiency and greater effect on viral levels based on immunofluorescence imaging results. The qRT-PCR analysis confirmed the high content imaging data, showing significant reduction in viral levels by all the selected siRNAs except for HSPA8 siRNA, which also failed to show viral reduction in the imaging screen assay (Figure 8B). Together, these data suggest that HSPA5, HSP90AB1, CCT6a and CCT2 function as important host factors during RVFV infection. To further assess chaperone function in RVFV infection, known chemical inhibitors of specific heat shock proteins identified by siRNA screening and qRT-PCR analysis (Figure 8) were analyzed in cell based infection assays (Figure 9A). Consistent with siRNA screening and qRT-PCR results, these inhibitors (HSP90 specific inhibitor 17-AAG, and HSP70 inhibitors KNK437 and BAPTA-AM) were able to greatly inhibit RVFV replication, without cytotoxic effects. We have also observed significant reduction in viral titers following HSP inhibitor treatment of HepG2 cells (Figures 9C–D, and data not shown), demonstrating that the observed effects are not cell type specific.

### Time of Addition and Real Time HSP Inhibition Studies

The HSP siRNA and inhibition data can be considered within two contexts: effects related to virus-host interaction in infected cells, and effects on functional integrity or structural characteristics of released virions. To address the former, we performed time-of-addition analysis of MP-12-infected HepG2 cells using HSP inhibitors 17-AAG and BAPTA-AM. Vero cells were infected with RVFV for 1 hour and after removal of the virus the inhibitors were added at specific times post infection (2 hr, 4 hr, 8 hr, or 14 hr p.i.) and their inhibitory effects on viral levels were measured by qRT-PCR (Figure 9B). The results show that addition of inhibitors several hours after infection and removal of the virus can lead to significant reduction of viral titers as compared to the DMSO-treated control (e.g, viral titer of 1.79×10^7^ for the DMSO control at the 2 hr time point compared to 3×10^5^ for 17-AAG and 4.66×10^4^ for BAPTA-AM, and viral titer of 2.57×10^7^ for the DMSO control at the 4 hr time point compared to 5.84×10^5^ for 17-AAG and 6.65×10^5^ for BAPTA-AM). In addition, greater reduction of viral levels is observed at earlier times (2 hr and 4 hr p.i.) compared to the effect observed if the inhibitors are added a few hours later (8 hr, or 14 hr p.i). Thus, at the 8 hr time point, the viral titers for the DMSO control, 17-AAG treatment, and BAPTA-AM treatment were 1.66×10^7^, 2.36×10^6^, and 4.85×10^6^ respectively, and at the 14 hr time point the viral titers for the DMSO control, 17-AAG treatment, and BAPTA-AM treatment were 2.72×10^7^, 1.65×10^7^, and 2×10^7^ respectively. These values show a significantly less reduction in viral titers for the 17-AAG and BAPTA-AM treatments at these time points when comparing with viral titers obtained for these inhibitor treatments at the 2 hr and 4 hr time points, which were presented above. We also performed plaque assay of the DMSO-treated and 17-AAG-treated samples from the 4 hr p.i. time point and, similar to the qRT-PCR results, observed significantly lower viral titer levels in the 17-AAG treated sample (data not shown). These observations suggested that the HSP effects are post viral entry and are more pronounced during the early infection phase in which significant viral replication/transcription occurs. To further confirm these conclusions, we also performed a real time study in which the effect of HSP inhibition with 17-AAG or BAPTA-AM on the levels of a luciferase-expressing derivative of MP-12 strain (rMP12-rLuc) was analyzed. This was accomplished by measuring the levels of luciferase signal post HSP inhibitor treatment followed byrMP12-rLuc infection. The measurements were made immediately upon reaching the 4 hour and 8 hour post infection times. We also included treatment of cells with a combination of 17-AAG and BAPTA-AM under conditions that did not induce cell toxicity. The results show a significant decrease in viral replication as early as 4 hours post-infection using a combination of 17-AAG and BAPTA-AM, and at 8 hours post-infection with 17-AAG alone. These results confirm the post viral entry effects of HSP inhibition during the early infection time period reported to correspond to the active replication/transcription phase [30].

### Potential Effects on Function or Structure of Virions

**Figure 10:**
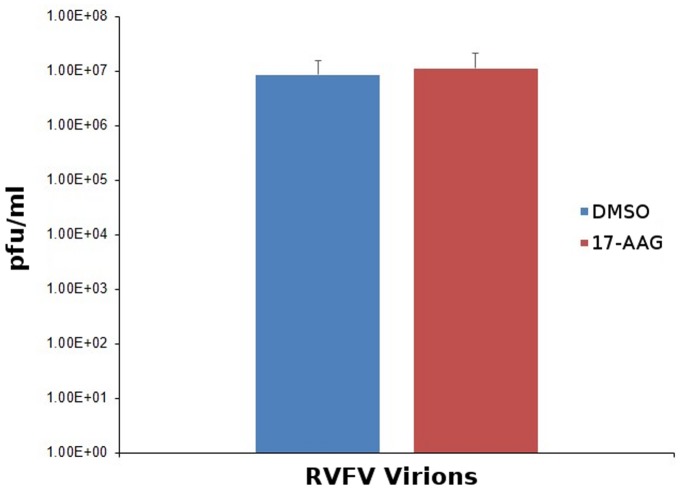
Analysis of infectivity of RVFV virions obtained under conditions of HSP inhibition. Vero cells were pretreated for 2 hours with equivalent volumes of either DMSO (control) or 17-AGG prior to infection with the MP-12 strain of RVFV (MOI 0.1). Following infection, cells were washed with PBS and media containing either DMSO or 17-AAG was added back to the cells. Twenty-four hours later, culture supernatants were collected and viral RNA levels were analyzed by qRT-PCR to measure the amounts of released virus. The supernatant samples were also functionally analyzed by plaque assays as described in the Methods section, and the plaque data was normalized to the amount of released virus based on the q-RT-PCR results. Data are average values from three independent assays.

To analyze potential effects of HSP inhibition on the functional ability of released virions, we compared culture supernatant obtained 24 hours after RVFV infection between untreated Vero cells and the same batch of cells treated with 17-AAG. For this analysis, both qRT-PCR assay to measure the amounts of released virus and plaque assays to measure infectivity were performed, and the plaque data was normalized to the amount of released virus based on the q-RT-PCR results. The results show that the functional ability of the virions is not compromised under conditions of strong HSP inhibition even though total viral levels are dramatically reduced (Figure 10), suggesting that while HSPs play an important role during viral production they do not necessarily function within the virions to affect infectivity. To investigate potential effects of HSP inhibition on structural features of the virions, we repeated the BLUE Native PAGE analysis following treatment with select siRNA constructs that showed strong viral inhibition (Figure 8). For this analysis (Figure S2), in addition to the scrambled siRNA and GAPDH siRNA controls (lanes 1 and 2 respectively), CCT6A 2545, HSPA5 6980, HSP90AB1 6999, and CCT2 20756 were used. As anticipated based on the results of the chaperone siRNA and inhibitor studies (Figure 8 and Figure 9), the overall protein band intensities in the HSP knockdown lanes are lower compared to the scrambled siRNA and GAPDH siRNA control lanes, because significantly less virions are recovered under the HSP knockdown conditions. However, comparison of the HSP siRNA lanes with the lanes for scrambled siRNA or GAPDH siRNA control (Figure S2) shows a lack of discernible effects on either the relative size or the relative abundances of the protein complex bands with respect to one another, suggesting that the structural features of released virions are not affected with respect to the host protein complexes that show virion association.

## Discussion

In this study, two complementary analytical methodologies were used to increase the depth and breadth of our understanding of RVFV by analyzing the host cell protein complement of purified RVFV virions. Gel LC/MS/MS methodology, the most widely used virion proteomic technique, was used in conjunction with another technique that has been traditionally used to study mitochondrial systems and allows isolation of protein complexes by Blue native electrophoresis [23]–[26]. The study produced an extensive list of virion-associated host proteins, and, as expected, viral proteins were also detected (Tables S1–S5 in File S1). We found multiple native protein complexes composed of host proteins and the RVFV nucleocapsid protein N. Bioinformatic analysis annotated many interactions within these complexes, leading credence to their authenticity; we plan to perform further functional characterizations of these complexes in future experiments. We discuss below the results presented here in support of the functional relevance of specific HSPs, which were among the overrepresented groups within our data set. Furthermore, although future studies are necessary to characterize all of the identified proteins and determine which are specifically significant to the process of infection, the coincidence of certain molecules with shared function makes it appropriate at this time to discuss these proteins in the context of RVFV as well.

### Cytoskeleton-associated Proteins, Integrins, and Cellular Entry Pathways

Our data strongly suggest that components of the host actin cytoskeleton associate with RVFV particles. Perhaps, these results are not surprising as associations with the actin cytoskeleton are among the most well understood host/virus interactions [38]–[40]. In fact, RVFV has been shown to cause disruptions in the cytoskeletal networks of polarized epithelial cells enabling viral entry and egress from both cell surfaces [41]. In addition, Fontana *et al*. observed actin in “viral tubes” in Golgi-associated viral factories for the model Bunyavirus, bunyamwera virus, indicating a role of actin in viral budding [42].

Cytoskeletal proteins identified in this study include a fairly comprehensive collection of proteins involved in multiple aspects of the cytoskeleton (e.g., assembly proteins including Talin 1 and the filament cross-linking protein Filamin A). Potentially, these interactions could constitute a signal transduction network where activities on the surface of the virion, such as binding events, could be relayed through actin filaments. Integrins are plasma membrane spanning proteins involved in cell/cell contacts and interactions with the extracellular matrix [43]–[45]. Integrins are only functional as heterodimeric complexes composed of an alpha and beta subunit. Integrins of both types were detected in association with RVFV virions in complex IV (Figures 3 and 5), and multiple additional alpha (1, 3 and 5) and beta (1 and 3) subunits through LC/MS/MS (Figure 2, Table S1 in File S1); integrin subunits have also been detected in HIV and influenza virions [11], [16]. Using databases of known protein-protein interactions, a seven node “Integrin-associated” network can be constructed within complex IV, consisting of three integrins (beta 1, alpha 1 and alpha 3), two proteins that regulate integrin function (the tetraspanin CD151 and galectin-8), and two proteins that are associated with the actin cytoskeleton (filamin C and actinin alpha 4) (Figure 6). Further, IPA analysis identified “integrin signaling” as an over-represented canonical pathway among the complete data set (Figure 6). Though there is limited data regarding the roles that host integrins play in virions, they have been shown to be the cellular receptors for multiple viruses such as West Nile virus, foot and mouth virus, hantavirus and human metapneumovirus [46]–[49]. Additionally, CD 151 associates with integrins to form tetraspanin-enriched microdomains that are important for viral assembly and budding [50]. The protein constituents and spatial orientation of this network suggest that this integrin-associated complex could play a role in virion entry or egress.

Consistent with high enrichment of integrins and actin/cytoskeletal in our data set, bioinformatic analysis of the results identified three over-represented canonical pathways that pertain to viral entry (“Caveolar-mediated endocytosis signaling”, “Virus entry via endocytic pathways”, and “Clathrin-mediated endocytosis signaling”) (Figure 6B; Table 2). In addition to integrins and cytoskeletal components, members of the Ras superfamily also populate nodes in each network (Table 2). The fact that known viral entry factors are associated with RVFV virions suggests that these proteins participate in different aspects of viral entry and could provide clues about molecular mechanisms. Interestingly, in support of our proteomics data, it was recently demonstrated that RVFV entry does involve caveola-mediated endocytosis [51]. Future experiments can build on these observations by silencing these putative host factors and other characteristic endosomal signaling protein to further elucidate RVFV entry mechanisms.

**Table 2:** RVFV virion-associated host proteins represented in canonical entry pathways.

Protein Function/Classification	Caveolar-mediatedEndocytosis Signaling	Virus Entry viaEndocytic Pathways	Clathrin-mediatedEndocytosis Signaling
Cytoskeleton	ACTB	ACTB	ACTB
Cytoskeleton	FLNA	FLNA	ARPC4
Cytoskeleton	FLNC	FLNC	X
Integrin-Associated	HLA-B	HLA-B	ITGB1
Integrin-Associated	ITGA1	ITGA1	ITGB3
Integrin-Associated	ITGA3	ITGA3	X
Integrin-Associated	ITGAV	ITGB1	X
Integrin-Associated	ITGB1	ITGB3	X
Integrin-Associated	ITGB3	X	X
Vesicular Transport	RAB5C	RAC1	RAB11A
Vesicular Transport	COPB1	CDC42	RAB5C
Vesicular Transport	X	X	RAB7A
Vesicular Transport	X	X	RAC1
Vesicular Transport	X	X	CDC42
Coat Component	X	CLTCL1	CLTCL1
Vesicle Disassembly	X	X	HSPA8
Coagulation	X	X	F2
Receptor Tyrosine Kinase	X	X	EPHB2

### Chaperones

Chaperones catalyze the proper folding of polypeptide chains into three dimensional structures and play central roles in coordinating cellular responses to unfolded protein stress. Chaperones from the heat shock protein HSP 70 and HSP 90 families have been consistently identified in viruses [11], [13], [14], [18]. In our analysis of RVFV virions, we found multiple heat shock proteins from these families and subunits of the T-complex protein (TCP) chaperone, CCT2 and CCT6A (Tables S1–S4 in File S1). Interestingly, to our knowledge, the association of CCT6A with virions of other viruses has not been reported and it is of interest to investigate whether this association may be unique to RVFV. Due to their apparent enrichment, siRNA screens of virion-associated host chaperones were conducted and the results indicated functional roles during RVFV infection for HSPA5 (BiP), HSPA8, HSP90AB1, and TCP subunits CCT6a and CCT2. Furthermore, inhibition studies using well-characterized small molecule HSP inhibitors demonstrated important functional roles for HSPs during RVFV infection, including members of the HSP90 and HSP70 families. The HSP association with the virions also prompted studies of potential effects on the virions themselves. However, we did not observe an adverse effect on the functional integrity of released virions following HSP inhibition. In addition, BLUE Native PAGE analysis of virion-associated host protein complexes did not signify structural changes in the virions following HSP inhibition, although clearly further extensive structural and ultrastructural studies are warranted for a comprehensive analysis.

BiP and HSPA8 are both members of the HSP 70 protein family. However, interestingly, these proteins have disparate effects on RVFV infection (Figure 7). BiP seems to be serving as an important host factor as its down regulation mitigates RVFV infection. Recently, our group has shown that BiP functions in the same capacity for Ebola and Marburg viruses [18]. Conversely, HSPA8 seems to be playing an anti-viral role. These findings are consistent with previous studies showing that the cytosolic enzyme HSPA8 binds and sequesters essential viral proteins, preventing replication [52] whereas BiP, a resident ER protein, is essential for the replication of multiple viruses [18]. It is likely that inhibition of BiP prevents protein maturation and processing of viral proteins through ER-associated pathways. Thus, the sub-cellular localization of these enzymes may account for the observed differences. Given the dual function of HSP 70 proteins, we were interested to see if small molecule inhibitors of these proteins could function as anti-viral compounds. The compound KNK 437 blocks induction of chaperones from the HSP 70, 72 and 105 families [53]. Another compound, BAPTA-AM, is a calcium chelator that decreases expression of BiP mRNA [54]. Interestingly, both compounds are able to inhibit RVFV replication, showing potential for further anti-viral development.

HSP90 chaperones constitute the most abundant folding enzymes in the cytosol. The HSP90 chaperone family includes highly homologous isoyzmes that can be characterized based on cellular expression as class alpha or beta proteins. Class alpha proteins are induced by folding stress and class beta proteins are expressed constitutively. In our analysis of RVFV virions, both classes of HSP 90 enzymes were detected by every method examined (LC/MS/MS, Western blotting and Blue Native PAGE), demonstrating their abundance in association with RVFV virions. To ascertain the importance of both HSP90 class proteins, siRNA was used to decrease expression of HSP 90AB1 (constitutively expressed) and HSP 90AA1 (inducible form) during RVFV infection. Interestingly, only the constitutively expressed isoform (HSP90AB1) was important for RVFV, as down regulation of the inducible isoform (HSP90AA1) had no effect. Perhaps this result is explained by the network analysis findings (Figure 6A). HSP90AB1 forms a major hub interacting with multiple diverse proteins, and inhibition of this protein could potentially result in a collapse of this extensive host protein network that is associated with RVFV virions.

HSP90 chaperones are promising targets for host directed anti-viral development. Several small molecule HSP90 inhibitors including geldanamycin and its derivatives such as 17-AAG, which is in phase II clinical trials for cancer treatment, have been shown to reduce viral titers in cell culture models of vaccinia virus, influenza virus, vesicular stomatitis virus, several paramyxoviruses, La Crosse virus and hepatitis C virus [55]–[59]. Consistent with the broad spectrum anti-viral activity of these inhibitors, 17-AAG also very effectively inhibits RVFV replication (Figure 9). Our data demonstrate a post viral entry effect for 17-AAG and BAPTA-AM. These effects manifest as early as 4 hours post infection, corresponding to a time window of very active viral replication and transcription [60]. In support of this conclusion, we have performed time course studies that show significant reduction of both viral RNA levels and RVFV protein levels by 17-AGG as early as 4–6 hours post infection (Benedict A, Kehn-Hall K, Bavari S, and Hakami RM, unpublished observations). Furthermore, the finding that at least some of the HSP effects are related to the replication/transcription phase is consistent with the role of HSP90 in stabilizing the RNA polymerases of several negative strand viruses [59].

These data show the therapeutic potential of targeting host chaperones to combat RVFV infection. Of particular interest is the potential of repurposing select HSP90 inhibitors to treat RVF, considering that several HSP90 inhibitors have already been progressing through clinical trials for cancer treatment.

## Supporting Information

Figure S1 **Western blot analysis of the effects of host siRNA knockdown on target HSP protein levels.** HeLa cells were transfected with CCT2, CCT6, HSP90AB1, or HSPA8 siRNA constructs, and forty eight hours post transfection total cellular lysates were prepared and analyzed by Western blot. Antibodies specific for CCT2, CCT6, HSP90β, and HSPA8 were used. In addition, GAPDH levels were analyzed for every lane as a loading control, and are shown underneath each corresponding lane. Protein signal levels were determined using densitometry measurements and normalization for each lane to account for potential loading differences was achieved based on the densitometry values for GAPDH in the same lane.
(JPG)

Figure S2 **Analysis of virion-associated host protein complexes recovered under conditions of HSP knockdown**. siRNA treatments were performed against several of the HSP target genes (CCT6A, HSPA5, HSP90AB1, CCT2). Treatments with siRNA specific to GAPDH and with scramble siRNA were also included as negative control. For each target HSP with duplicate siRNA set, the siRNA construct with greatest knockdown efficiency and larger effect on viral titers was used. Following siRNA knockdown, cells were infected with MP-12 and culture supernatants from each condition were processed and analyzed by BLUE native PAGE. Lane designations are as follows: Lane 1: siRNA Control; Lane 2: siRNA GAPDH; Lane 3: CCT6A 2545; Lane 4: HSPA5 6980; Lane 5: HSP90AB1 6999; Lane 6: CCT2 20756.
(TIF)

File S1 **Supplementary Proteomic Data Tables.** Table S1: Proteins identified in RVFV virions. Table S2: Proteins identified in native complex 1. Table S3: Proteins identified in native complex 2. Table S4: Proteins identified in native complexes 3 and 4. Table S5: Common proteins between purified RVFV virions and non-infected control sample obtained by cell lysis and subjected to the same purification procedure as virions side by side.
(DOCX)
