# Supplementary figures and images for: Multi-Faceted Proteomic Characterization of Host Protein Complement of Rift Valley Fever Virus Virions and Identification of Specific Heat Shock Proteins, Including HSP90, as Important Viral Host Factors
Source: PLoS One. 2014 May 8;9(5):e93483. doi: 10.1371/journal.pone.0093483 (PMC4014464; doi:10.1371/journal.pone.0093483)

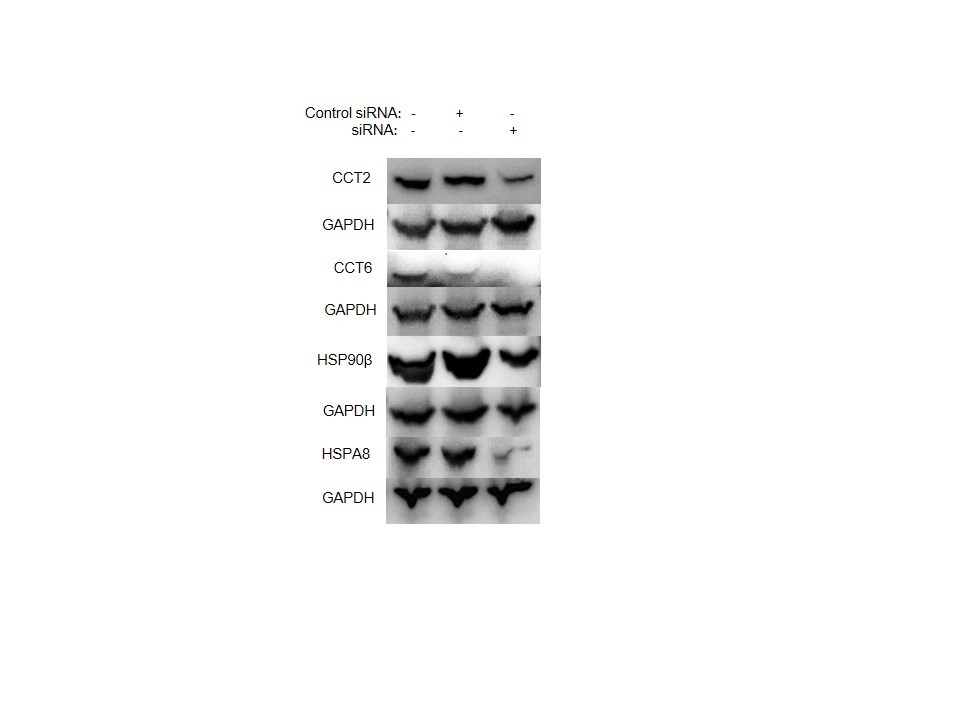

Supplement: Figure S1 — Western blot analysis of the effects of host siRNA knockdown on target HSP protein levels. HeLa cells were transfected with CCT2, CCT6, HSP90AB1, or HSPA8 siRNA constructs, and forty eight hours post transfection total cellular lysates were prepared and analyzed by Western blot. Antibodies specific for CCT2, CCT6, HSP90β, and HSPA8 were used. In addition, GAPDH levels were analyzed for every lane as a loading control, and are shown underneath each corresponding lane. Protein signal levels were determined using densitometry measurements and normalization for each lane to account for potential loading differences was achieved based on the densitometry values for GAPDH in the same lane. (JPG) [file pone.0093483.s001.jpg]

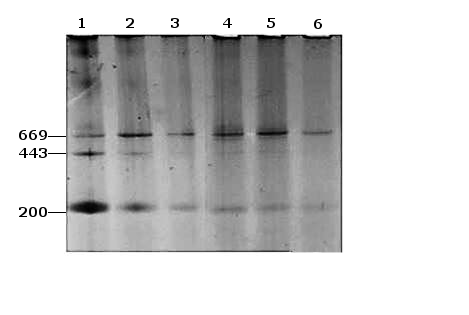

Supplement: Figure S2 — Analysis of virion-associated host protein complexes recovered under conditions of HSP knockdown. siRNA treatments were performed against several of the HSP target genes (CCT6A, HSPA5, HSP90AB1, CCT2). Treatments with siRNA specific to GAPDH and with scramble siRNA were also included as negative control. For each target HSP with duplicate siRNA set, the siRNA construct with greatest knockdown efficiency and larger effect on viral titers was used. Following siRNA knockdown, cells were infected with MP-12 and culture supernatants from each condition were processed and analyzed by BLUE native PAGE. Lane designations are as follows: Lane 1: siRNA Control; Lane 2: siRNA GAPDH; Lane 3: CCT6A 2545; Lane 4: HSPA5 6980; Lane 5: HSP90AB1 6999; Lane 6: CCT2 20756. (TIF) [file pone.0093483.s002.tif]
