# Supplementary material for: Multi-Faceted Proteomic Characterization of Host Protein Complement of Rift Valley Fever Virus Virions and Identification of Specific Heat Shock Proteins, Including HSP90, as Important Viral Host Factors
Source: PLoS One. 2014 May 8;9(5):e93483. doi: 10.1371/journal.pone.0093483 (PMC4014464; doi:10.1371/journal.pone.0093483)
Supplement: File S1 — Supplementary Proteomic Data Tables. Table S1: Proteins identified in RVFV virions. Table S2: Proteins identified in native complex 1. Table S3: Proteins identified in native complex 2. Table S4: Proteins identified in native complexes 3 and 4. Table S5: Common proteins between purified RVFV virions and non-infected control sample obtained by cell lysis and subjected to the same purification procedure as virions side by side. (DOCX) [file pone.0093483.s003.docx]

**Table S1.** Proteins identified in RVFV virions

| Protein Name | Excised Gel Areas | Accession Number | Protein MWT | Highest  Peptide  Count | Molecular Function |
| --- | --- | --- | --- | --- | --- |
| Embryo-specific fibronectin 1 transcript variant [Bos taurus] | 1-4, 6 | gi\|220028657 | 262 kDa | 85 | Extracellular matrix, cell adhesion |
| PREDICTED: similar to fibronectin 1 isoform 1 preproprotein [Macaca mulatta] | 1,2 | gi\|109100908 | 288 kDa | 81 | Extracellular matrix, cell adhesion |
| Fibronectin 1, isoform CRA_h [Homo sapiens] | 1-4 | gi\|119590943 (+5) | 256 kDa | 77 | Extracellular matrix, cell adhesion |
| Unnamed protein product [Mus musculus] | 1 | gi\|74188584 | 250 kDa | 38 | Unknown |
| Alpha-2-macroglobulin [Bos taurus] | 1-8,  10,11 | gi\|157954061 | 168 kDa | 35 | Protease inhibitor |
| PREDICTED: similar to alpha 3 type VI collagen, partial [Bos taurus] | 1 | gi\|119890597 | 178 kDa | 20 | Cell binding |
| Inter-alpha (globulin) inhibitor H4 (plasma Kallikrein-sensitive glycoprotein) [Bos taurus] | 1-12 | gi\|59857769 (+1) | 102 kDa | 33 | Protease inhibitor |
| Chain A, Trypsin In Complex With Borate | 1-12 | gi\|110590762 (+7) | 23 kDa | 27 | Protease |
| Collagen, type VI, alpha 3 [Canis lupus familiaris] | 1 | gi\|157151714 | 343 kDa | 13 | Cell binding |
| Keratin 1 [Homo sapiens] | 1-3,5-10, 12 | gi\|11935049 (+2) | 66 kDa | 22 | Cytoskeleton |
| Serum albumin | 1-12 | gi\|1351907 (+1) | 69 kDa | 30 | Carrier protein |
| PREDICTED: similar to fatty acid synthase [Macaca mulatta] | 1 | gi\|109119169 | 273 kDa | 12 | Fatty acid synthase activity |
| RecName: Full=Complement C3 | 1-10 | gi\|124056491 (+1) | 187 kDa | 37 | Immune response |
| Thrombospondin 1 [Bos taurus] | 1-4, 6-8, 10 | gi\|41386685 | 130 kDa | 29 | Cell adhesion |
| Filamin A, alpha isoform 1 [Homo sapiens] | 1,2 | gi\|116063573 (+10) | 280 kDa | 9 | Scaffolding, promotes actin branching |
| PREDICTED: similar to complement component 4A [Bos taurus] | 1-6,8-10 | gi\|119915491 | 193 kDa | 34 | Immune response |
| PREDICTED: keratin 25D isoform 4 [Pan troglodytes] | 1-4,6,  8-10 | gi\|114667507 (+8) | 100 kDa | 15 | Cytoskeleton |
| Keratin 2 [Pan troglodytes] | 1,10 | gi\|148727309 (+2) | 66 kDa | 8 | Cytoskeleton |
| PREDICTED: similar to Keratin, type I cytoskeletal 14 (Cytokeratin-14) (CK-14) (Keratin-14) (K14) [Pan troglodytes] | 1,2,6,8,  10,12 | gi\|114667176 | 107 kDa | 14 | Cytoskeleton |
| Myosin, heavy polypeptide 9, non-muscle [Homo sapiens] | 1,3,4 | gi\|12667788 (+1) | 227 kDa | 7 | Cytokinesis |
| Hemoglobin, gamma [Bos taurus] | 1-12 | gi\|62460494 | 16 kDa | 21 | Oxygen transport |
| PREDICTED: similar to plectin 1 isoform 3 [Macaca mulatta] | 1 | gi\|109087706 (+27) | 532 kDa | 5 | Cytoskeleton |
| Fibulin 1 [Bos taurus] | 1-4,6,11,  12 | gi\|148232266 | 78 kDa | 9 | Extracellular matrix component |
| Fer-1 like protein 3 [Homo sapiens] | 1,2 | gi\|10834587 (+7) | 233 kDa | 4 | Phospholipid binding |
| PREDICTED: heparan sulfate proteoglycan 2 [Macaca mulatta] | 1 | gi\|108998883 (+7) | 469 kDa | 4 | Cell binding, cell signaling |
| Genome polyprotein - Rift Valley fever virus | 1 | gi\|478819 (+9) | 237 kDa | 4 | Putative transcriptase replicase |
| Apolipoprotein A-I, apoA-1 [Bos=cattle, Friesian-Holstein male calves aged 2-4 weeks, Peptide, 247 aa] | 1-12 | gi\|245563 (+1) | 28 kDa | 32 | Lipid binding |
| M protein [Rift Valley fever virus] | 1-8,  10-12 | gi\|27881464 (+8) | 131 kDa | 33 |  |
| Serine proteinase inhibitor, clade A, member 1 [Bos taurus] | 1-10, 12 | gi\|27806941 | 46 kDa | 11 | Protease inhibitor |
| Complement factor H [Bos taurus] | 1-3 | gi\|76677897 | 140 kDa | 20 | Immune response |
| PREDICTED: laminin, gamma 1 isoform 3 [Pan troglodytes] | 1 | gi\|114568344 (+2) | 178 kDa | 3 | Cell differentiation |
| PREDICTED: von Willebrand factor [Bos taurus] | 1 | gi\|194667464 | 300 kDa | 3 | Extracellular Matrix Component |
| Alpha-2-HS-glycoprotein [Bos taurus] | 1-12 | gi\|27806751 | 38 kDa | 7 | Serum protein |
| Apolipoprotein E [Bos taurus] | 1,2,7,8 | gi\|110225417 (+2) | 27 kDa | 7 | Lipid binding |
| PREDICTED: talin 1 [Macaca mulatta] | 1 | gi\|109111052 (+27) | 261 kDa | 2 | Actin assembly |
| PREDICTED: similar to annexin A2 isoform 1 [Macaca mulatta] | 3-9 | gi\|109081460 (+7) | 54 kDa | 37 | Phospholipase inhibitor, cytoskeleton binding |
| PREDICTED: similar to Actin, cytoplasmic 2 (Gamma-actin) [Rattus norvegicus] | 2-12 | gi\|109492380 (+44) | 59 kDa | 23 | Cytoskeleton component |
| Nucleocapsid protein [Rift Valley fever virus] | 8-11 | gi\|6006977 (+1) | 27 kDa | 27 | Role in viral RNA synthesis |
| Factor V | 3,4, 6-8 | gi\|163040 (+1) | 248 kDa | 4 | Clotting factor |
| PREDICTED: Na+/K+ -ATPase alpha 1 subunit isoform 3 [Macaca mulatta] | 3,4 | gi\|109014549 (+1) | 113 kDa | 17 | Electrochemical gradient maintenance |
| Valosin-containing protein [Homo sapiens] | 3,4 | gi\|111305821 (+8) | 89 kDa | 31 | ATP binding, vesicle fusion |
| PREDICTED: pyruvate kinase 3 isoform 9 [Macaca mulatta] | 2,4-6 | gi\|109081748 (+20) | 65 kDa | 20 | Metabolism |
| Hemoglobin alpha chain [Bos taurus] | 6-12 | gi\|116812902 | 15 kDa | 8 | Oxygen transport |
| PREDICTED: glyceraldehyde-3-phosphate dehydrogenase [Macaca mulatta] | 7-9 | gi\|109095230 | 36 kDa | 19 | Metabolism |
| PREDICTED: enolase 1 isoform 5 [Macaca mulatta] | 6,7 | gi\|108996402 (+7) | 47 kDa | 20 | Ion binding, transcription factor activity |
| PREDICTED: annexin IV isoform 5 [Pan troglodytes], | 8,9 | gi\|114577902 | 36 kDa | 20 | Phospholipase inhibitor, cytoskeleton binding |
| VLA-3 alpha subunit [Homo sapiens] | 2,3,8 | gi\|220141 | 114 kDa | 19 | Cell surface adhesion |
| PREDICTED: similar to eukaryotic translation elongation factor 1 alpha 1 isoform 3 [Macaca mulatta] | 4,6-10 | gi\|109071714 (+36) | 49 kDa | 6 | Regulation of translation |
| Inter-alpha globulin inhibitor H2 polypeptide [Bos taurus] | 2-5 | gi\|148238273 | 106 kDa | 10 | Protease inhibitor |
| PREDICTED: heat shock 90kDa protein 1, beta  isoform 2 [Macaca mulatta] | 4,6 | gi\|109071319 (+15) | 80 kDa | 14 | Chaperone |
| PREDICTED: clathrin heavy chain 1 isoform 6 [Macaca mulatta] | 2 | gi\|109114529 (+21) | 192 kDa | 22 | Intracellular trafficking, endocytosis |
| PREDICTED: similar to C4b-binding protein alpha chain precursor (C4bp) (Proline-rich protein) (PRP) [Bos taurus] | 7,10-12 | gi\|76677514 | 22 kDa | 14 | Activation of complement cascade |
| Hypothetical protein [synthetic construct] | 2,3 | gi\|117645218 (+3) | 88 kDa | 17 | Unknown |
| PREDICTED: annexin I isoform 1 [Macaca mulatta] | 6-8 | gi\|109111790 (+4) | 39 kDa | 11 | Phospholipase inhibitor, cytoskeleton binding |
| RecName: Full=Vimentin | 7,10,11 | gi\|75075845 | 54 kDa | 13 | Cytoskeleton component |
| PREDICTED: similar to histone cluster 1, H2ag [Sus scrofa] | 9-12 | gi\|194039792 | 48 kDa | 6 | Chromatin, DNA binding |
| Tubulin, beta 2C | 6 | gi\|118404276 (+8) | 50 kDa | 16 | Microtubule component |
| PREDICTED: aldehyde dehydrogenase 1A1 isoform 5 [Macaca mulatta] | 6,7 | gi\|109111776 (+4) | 55 kDa | 15 | Metabolism |
| Unnamed protein product [Macaca fascicularis] | 5,8 | gi\|90075592 (+1) | 76 kDa | 14 | Unknown |
| Transferrin [Bos taurus] | 4,5 | gi\|114326282 (+1) | 78 kDa | 13 | Iron binding/transport |
| PREDICTED: annexin A11 [Macaca mulatta] | 6,7 | gi\|109089204 (+1) | 54 kDa | 14 | Phospholipase inhibitor, cytoskeleton binding |
| Complement component 4 binding protein, alpha chain precursor [Bos taurus] | 4 | gi\|146231832 (+1) | 69 kDa | 15 | Activation of complement cascade |
| Heat shock cognate 71 kDa protein | 4,5,7 | gi\|123647 (+32) | 71 kDa | 8 | Chaperone |
| Immunoglobulin heavy chain constant region [Bos taurus] | 4 | gi\|34538498 | 50 kDa | 14 | Immune response |
| Annexin A5 | 8 | gi\|75075702 (+1) | 36 kDa | 17 | Phospholipase inhibitor, cytoskeleton  binding |
| Unnamed protein product [Macaca fascicularis] | 4,5 | gi\|90075134 | 58 kDa | 8 | Unknown |
| PREDICTED: tubulin, alpha, ubiquitous isoform 21 [Macaca mulatta] | 2,6,7 | gi\|109096460 (+37) | 49 kDa | 8 | Microtubule component |
| PREDICTED: lactate dehydrogenase A isoform 2 [Macaca mulatta] | 7,8 | gi\|109107094 (+3) | 37 kDa | 9 | Metabolism |
| Fibrinogen beta chain [Bos taurus] | 5-7 | gi\|218931172 | 56 kDa | 5 | Clotting factor |
| PREDICTED: complement component 5 [Bos taurus] | 3,4 | gi\|119901003 | 189 kDa | 9 | Activation of complement cascade |
| PREDICTED: similar to phosphoglycerate kinase 1 isoform 4 [Macaca mulatta] | 7 | gi\|109131308 (+7) | 45 kDa | 13 | Metabolism |
| T-complex protein 1 subunit beta [Homo sapiens] | 6 | gi\|197692147 (+2) | 57 kDa | 13 | Chaperone, ATP binding |
| Chain A, Refined Solution Structure of Human  Profilin I | 11,12 | gi\|157833469 (+5) | 15 kDa | 8 | Regulation of actin polymerization |
| Chain A, Human Adp-Ribosylation Factor 1 Complexed with Gdp, Full Length Non-Myristoylated | 10,11 | gi\|1065361 (+18) | 21 kDa | 9 | Nucleotide binding, vesicular trafficking |
| Chain A, Crystal Structure of the Heterodimeric Complex of Human Rgs1 And Activated Gi Alpha 1 | 7 | gi\|109158062 (+8) | 37 kDa | 10 | Regulation of G-protein mediated signaling |
| PREDICTED: complement component 4A [Bos taurus] | 2,4 | gi\|119915494 | 193 kDa | 25 | Activation of complement cascade |
| PREDICTED: similar to myristoylated alanine-rich protein kinase C substrate [Macaca mulatta] | 3,4 | gi\|109072985 | 31 kDa | 6 | Regulation of actin polymerization |
| RecName: Full=Prothrombin | 4,8 | gi\|135806 (+2) | 71 kDa | 4 | Coagulation factor |
| PREDICTED: similar to basigin isoform 1 [Macaca mulatta] | 5,6 | gi\|109122669 | 52 kDa | 5 | Stimulation of matrix metalloprotease activity |
| PREDICTED: similar to annexin VII isoform 2 isoform 1 [Macaca mulatta] | 6-8 | gi\|109089308 (+2) | 53 kDa | 6 | Phospholipase inhibitor, cytoskeleton binding |
| PREDICTED: similar to non-metastatic cells 1, protein (NM23A) expressed in isoform a [Macaca mulatta] | 10,11 | gi\|109114364 (+4) | 27 kDa | 6 | Nucleotide binding, magnesium binding |
| Collagen, type VI, alpha 1 [Bos taurus] | 2 | gi\|219804724 | 109 kDa | 8 | Cell binding |
| RecName: Full=Glucose-6-phosphate isomerase | 5,6 | gi\|75075955 (+1) | 63 kDa | 5 | Metabolism |
| S-adenosylhomocysteine hydrolase [Bos taurus] | 6,7 | gi\|77735583 | 48 kDa | 5 | Hydrolytic enzyme, regulates S-adenosylhomo-cysteine |
| PREDICTED: aldolase A isoform 3 [Macaca mulatta], gi\|109128142\|ref\|XP_001108119.1\|  PREDICTED: aldolase A isoform 4 [Macaca mulatta], gi\|109128144\|ref\|XP_001108169.1\|  PREDICTED: aldolase A isoform 5 [Macaca mulatta], gi\|109128146\|ref\|XP_001108223.1\|  PREDICTED: aldolase A isoform 6 [Macaca mulatta], gi\|109128148\|ref\|XP_001108269.1\|  PREDICTED: aldolase A isoform 7 [Macaca mulatta], gi\|109128150\|ref\|XP_001108325.1\|  PREDICTED: aldolase A isoform 8 [Macaca mulatta], gi\|109128152\|ref\|XP_001108381.1\|  PREDICTED: aldolase A isoform 9 [Macaca mulatta], gi\|109128154\|ref\|XP_001108440.1\|  PREDICTED: aldolase A isoform 10 [Macaca mulatta], gi\|90078570\|dbj\|BAE88965.1\| | 7 | gi\|109128140 (+7) | 39 kDa | 9 | Metabolism |
| Unnamed protein product [Homo sapiens] | 3 | gi\|194380758 (+4) | 125 kDa | 10 | Unknown |
| PREDICTED: similar to factor activating exoenzyme S [Ornithorhynchus anatinus] | 9 | gi\|149637731 (+5) | 38 kDa | 10 | Mediator of trans-membrane signaling |
| Cystathionine gamma-lyase [Macaca fascicularis] | 7 | gi\|58373388 (+1) | 45 kDa | 10 | Synthetic enzyme in trans-sulfuration pathway |
| Apolipoprotein H (beta-2-glycoprotein I) [Bos taurus] | 5 | gi\|109939993 (+4) | 38 kDa | 3 | Lipoprotein metabolism, negative surface binding protein |
| Flagellin domain-containing protein [Sinorhizobium medicae WSM419] | 7,8 | gi\|150395493 (+3) | 41 kDa | 4 | Cell motility |
| Chain C, The Crystal Structure of Modified Bovine Fibrinogen | 3,6 | gi\|6980816 (+1) | 47 kDa | 6 | Coagulation |
| PREDICTED: similar to Cofilin-1 (Cofilin, non-muscle isoform) | 10,11 | gi\|109105452 (+2) | 49 kDa | 6 | Actin binding protein |
| PREDICTED: similar to peroxiredoxin 1 isoform 2 [Macaca mulatta] | 9,10 | gi\|109003875 (+12) | 22 kDa | 6 | Peroxidase activity, oxidative stress response |
| PREDICTED: aldo-keto reductase family 1, member B1 [Macaca mulatta] | 8 | gi\|109068261 (+1) | 36 kDa | 8 | Metabolism |
| PREDICTED: CD63 molecule isoform 1 [Macaca mulatta] | 6-8 | gi\|109097131 (+8) | 23 kDa | 3 | Integrin binding |
| EPH receptor A2 [Macaca mulatta] | 3,7 | gi\|100818625 (+7) | 108 kDa | 5 | Receptor tyrosine kinase, signaling |
| Clusterin [Bos taurus] | 7,8 | gi\|27806907 | 51 kDa | 6 | Cell binding, apoptosis |
| PREDICTED: integrin alpha-V [Macaca mulatta] | 3,9 | gi\|109100308 | 116 kDa | 6 | Integral membrane receptor |
| PREDICTED: IQ motif containing GTPase activating protein 1 [Pan troglodytes] | 2,4 | gi\|114659004 (+4) | 194 kDa | 5 | Regulates reorganization of actin cytoskeleton |
| PREDICTED: similar to Alpha-2-macroglobulin precursor (Pregnancy zone protein) (Alpha-2-M), partial [Bos taurus] | 2 | gi\|119893044 | 117 kDa | 7 | Protease inhibitor |
| RecName: Full=Triosephosphate isomerase | 9 | gi\|136062 | 27 kDa | 8 | Metabolism |
| PREDICTED: similar to actinin, alpha 4 isoform 2 [Macaca mulatta] | 3 | gi\|109124595 (+25) | 104 kDa | 8 | Actin crosslinking |
| PREDICTED: prostaglandin F2 receptor negative regulator [Macaca mulatta] | 3 | gi\|109014719 | 117 kDa | 8 | Regulator of G-protein mediated signaling |
| Vitronectin [Bos taurus] | 4-6 | gi\|78045497 | 54 kDa | 3 | Cell adhesion factor |
| Eukaryotic translation elongation factor 1 gamma [Homo sapiens] | 6 | gi\|15530265 (+12) | 50 kDa | 4 | Regulation of translation |
| PREDICTED: similar to Brain abundant, membrane attached signal protein 1 [Pan troglodytes] | 6-8 | gi\|114599052 (+1) | 43 kDa | 3 | Transcription factor |
| Multidrug resistance p-glycoprotein [Macaca fascicularis] | 2,3 | gi\|31442763 (+2) | 142 kDa | 3 | Transport of substances across plasma membrane |
| RecName: Full=Antithrombin-III | 4,5 | gi\|109940161 (+2) | 52 kDa | 4 | Protease inhibitor, clotting inhibitor |
| PREDICTED: hypothetical protein isoform 1 [Pan troglodytes] | 11,12 | gi\|114686339 (+4) | 14 kDa | 5 | Unknown |
| PREDICTED: moesin [Macaca mulatta] | 4 | gi\|109131058 (+1) | 102 kDa | 5 | Actin binding |
| Chain X, Crystal Structure of Akr1b10 Complexed With Nadp+ and Tolrestat | 8 | gi\|119388973 (+4) | 36 kDa | 6 | Metabolism |
| Arginine deiminase [Mycoplasma arginini] | 6 | gi\|148361417 (+5) | 44 kDa | 6 | Protein post-translational modification |
| PREDICTED: similar to Ras-related protein Rap-1b precursor (GTP-binding protein smg p21B) [Equus caballus] | 10 | gi\|149715267 (+8) | 21 kDa | 7 | Signaling, GTPase |
| PREDICTED: T-complex protein 1 isoform 6 [Macaca mulatta] | 5 | gi\|109073165 (+4) | 60 kDa | 7 | Chaperone |
| PREDICTED: similar to chaperonin containing TCP1, subunit 8 (theta) isoform 5 [Macaca mulatta] | 5 | gi\|109065591 (+1) | 60 kDa | 7 | Chaperone |
| PREDICTED: similar to Myosin light polypeptide 6 | 11 | gi\|109067280 (+21) | 17 kDa | 7 | Cytokinesis |
| PREDICTED: complement component 1, q subcomponent, C chain isoform 1 [Bos taurus] | 9 | gi\|194665024 (+1) | 26 kDa | 7 | Activation of complement cascade |
| Fibrinogen alpha chain [Bos taurus] | 12 | gi\|148745450 (+2) | 67 kDa | 2 | Coagulation |
| PREDICTED: heterogeneous nuclear ribonucleoprotein A1 isoform 1 [Macaca mulatta] | 10 | gi\|109098434 (+65) | 35 kDa | 2 | RNA binding |
| Ig heavy chain precursor (B/MT.4A.17.H5.A5) | 6 | gi\|108750 (+2) | 51 kDa | 2 | Immune response |
| PREDICTED: similar to KIAA0120 [Pan troglodytes] | 10,11 | gi\|114560493 (+12) | 47 kDa | 4 | Unknown |
| Epithelial cell adhesion molecule [Macaca mulatta] | 7,8 | gi\|91064856 | 35 kDa | 3 | Cell adhesion |
| PREDICTED: similar to plasma membrane calcium ATPase 1 isoform 1b isoform 2 [Macaca mulatta] | 2,3 | gi\|109098172 (+53) | 131 kDa | 3 | Calcium homeostasis, transport |
| GTP-binding regulatory protein Gs alpha-XL chain – rat | 6,7 | gi\|1086315 (+39) | 92 kDa | 4 | Nucleotide binding, signal transduction |
| PREDICTED: similar to Ras-related protein Rab-5C (RAB5L) (L1880) [Equus caballus] | 9 | gi\|149723790 (+3) | 23 kDa | 6 | Nucleotide binding, signal transduction |
| Complement component 1, q subcomponent, B chain [Bos taurus], | 9 | gi\|114051157 | 26 kDa | 6 | Activation of complement cascade |
| PREDICTED: similar to integrin beta chain, beta 3 precursor isoform 2 [Macaca mulatta] | 3 | gi\|109116527 | 87 kDa | 6 | Cellular receptor |
| PREDICTED: similar to 90-kDa heat shock protein [Monodelphis domestica] | 4 | gi\|126290220 (+13) | 85 kDa | 12 | Chaperone |
| Peptidylprolyl isomerase A [Homo sapiens] | 11 | gi\|10863927 (+19) | 18 kDa | 6 | Protein folding |
| Glucose transporter type 1 [Feliscatus] | 6 | gi\|108742763 (+33) | 40 kDa | 2 | Glucose transport |
| PREDICTED: similar to 40S ribosomal protein SA (p40) | 7,9 | gi\|109041466 (+35) | 33 kDa | 2 | Ribosome component, receptor for laminin |
| Unnamed protein product [Homo sapiens] | 8 | gi\|10436857 (+14) | 71 kDa | 3 | Unknown |
| PREDICTED: CD44 antigen isoform 8 [Macaca mulatta] | 10 | gi\|109106764 (+11) | 81 kDa | 3 | Receptor for hyaluronic acid, cell-cell interactions |
| Kappa-B motif-binding phosphoprotein – mouse | 7,8 | gi\|1083569 (+43) | 51 kDa | 3 | Ribonucleotide binding |
| PREDICTED: similar to eukaryotic initiation factor 4AI [Monodelphis domestica] | 6,7 | gi\|126309196 (+21) | 52 kDa | 3 | Transcription regulation |
| PREDICTED: plexin B2 [Macaca mulatta] | 2,9 | gi\|109094648 | 204 kDa | 3 | Receptor for SEMA4D, actin cytoskeleton regulation |
| PREDICTED: similar to RAB11a, member RAS oncogene family [Macaca mulatta] | 9,10 | gi\|109081641 (+83) | 34 kDa | 3 | Secretory pathway regulator |
| Large erk/cek5 tyrosine kinase [Homo sapiens] | 6,7 | gi\|1060895 (+31) | 54 kDa | 3 | Receptor tyrosine kinase activity |
| Ferritin heavy chain [Felis catus] | 10 | gi\|114326408 (+17) | 21 kDa | 3 | Iron ion binding |
| PREDICTED: similar to ras-related C3 botulinum toxin substrate 1 isoform Rac1b [Macaca mulatta] | 10 | gi\|109065949 (+38) | 23 kDa | 4 | Nucleotide binding, cytoskeleton regulation |
| PREDICTED: similar to chaperonin containing TCP1, subunit 4 (delta) [Macaca mulatta] | 5 | gi\|109103069 (+18) | 54 kDa | 4 | Chaperone |
| PREDICTED: stomatin isoform 2 [Macaca mulatta] | 8 | gi\|109110361 (+17) | 32 kDa | 4 | Regulation of ion movement |
| PREDICTED: hypothetical  protein [Pan troglodytes] | 8 | gi\|114550604 (+28) | 37 kDa | 4 | Unknown |
| Inter-alpha (globulin) inhibitor H3 [Bos taurus] | 4 | gi\|110331845 (+1) | 99 kDa | 4 | Protease inhibitor |
| Chain A, The Crystal Structure of Human Hypoxanthine-Guanine Phosphoribosyltransferase with Bound Gmp | 9 | gi\|1065265 (+37) | 24 kDa | 5 | Purine synthesis |
| PREDICTED: similar to 14.3.3 protein [Pan troglodytes] | 9 | gi\|114576225 (+12) | 45 kDa | 6 | Mediator of phosphoserine signaling |
| Unnamed protein product [Homo sapiens] | 5 | gi\|194373693 (+10) | 69 kDa | 5 | Unknown |
| unnamed protein product [Homo sapiens] | 6 | gi\|194385108 (+4) | 52 kDa | 5 | Unknown |
| Tyrosine 3-monooxygenase/tryptophan 5-monooxygenase activation protein, eta polypeptide [Bostaurus] | 9 | gi\|73853758 | 28 kDa | 6 | Actin binding; insulin-like growth factor receptor binding |
| PREDICTED: similar to Myosin regulatory light chain 2, nonsarcomeric (Myosin RLC) isoform 2 [Macaca mulatta] | 10 | gi\|109121665 (+21) | 20 kDa | 5 | Motor protein |
| PREDICTED: guanine nucleotide binding protein (G protein), alpha 11 (Gq class) [Macaca mulatta] | 7 | gi\|109122902 (+4) | 46 kDa | 5 | Transmembrane signaling mediator |
| PREDICTED: coagulation factor XIII, A1 polypeptide [Bos taurus] | 4 | gi\|119915840 | 83 kDa | 5 | Coagulation |
| PREDICTED: aquaporin 1 isoform 2 [Pan troglodytes] | 9 | gi\|114612688 (+8) | 34 kDa | 2 | Regulation of cellular osmotic pressure |
| group-specific component (vitamin D binding protein) [Bos taurus] | 5 | gi\|78369364 | 53 kDa | 2 | Vitamin D binding, actin binding |
| RecName: Full=CD9 antigen; AltName: Full=27 kDa diphtheria toxin receptor-associated protein | 9,10 | gi\|231723 | 25 kDa | 2 | Cellular adhesion |
| Immunoglobulin lambda-like polypeptide 1 [Bos taurus], | 9 | gi\|139948632 (+7) | 25 kDa | 3 | B-cell development |
| RecName: Full=Kininogen-1 | 5 | gi\|125505 (+2) | 69 kDa | 2 | Protease inhibitor, blood coagulation |
| Ras-related protein Rab-10, | 10 | gi\|464553 | 23 kDa | 3 | Nucleotide binding |
| PREDICTED: similar to plasminogen activator inhibitor-1 isoform 2 [Macaca mulatta] | 7 | gi\|109066173 (+1) | 45 kDa | 3 | Protease inhibitor |
| PREDICTED: Rho GDP dissociation inhibitor (GDI) alpha isoform 1 [Macaca mulatta] | 9 | gi\|109119112 (+6) | 26 kDa | 3 | Regulates GTP/GDP exchange of Rho proteins |
| Tubulin, beta [Homo sapiens] | 6 | gi\|18088719 (+9) | 50 kDa | 14 | Microtubule component |
| EH domain-containing protein FKSG7 [Homo sapiens] | 5 | gi\|11066968 (+4) | 61 kDa | 3 | Endosomal transport |
| Lactate dehydrogenase B variant 1 [Bos grunniens] | 8 | gi\|116282345 (+2) | 37 kDa | 6 | Metabolism |
| PREDICTED: chaperonin containing TCP1, subunit 5 (epsilon) isoform 2 [Macaca mulatta] | 5 | gi\|109076725 (+17) | 53 kDa | 4 | Chaperone |
| PREDICTED: similar to 3-hydroxybutyrate dehydrogenase, type 2 [Macaca mulatta] | 9 | gi\|109075205 | 27 kDa | 4 | Metabolism |
| PREDICTED: similar to aminopeptidase puromycin sensitive, partial [Macaca mulatta] | 3 | gi\|109114102 (+9) | 91 kDa | 4 | Exopeptidase |
| Ras homolog gene family, member A [Homo sapiens] | 10 | gi\|10835049 (+19) | 22 kDa | 4 | Regulates endosomal pathway |
| MHC class I antigen [Macaca mulatta] | 7 | gi\|161087572 (+1) | 40 kDa | 4 | Antigen presentation |
| PREDICTED: tyrosine 3-monooxygenase/tryptophan 5-monooxygenase activation protein, beta polypeptide [Macaca mulatta] | 9 | gi\|109091960 (+15) | 28 kDa | 5 | Phosphoserine motif binding, signal transduction |
| PREDICTED: 2',3'-cyclic nucleotide 3' phosphodiesterase [Pan troglodytes] | 7 | gi\|114667114 (+5) | 59 kDa | 4 | Nucleotide metabolism |
| PREDICTED: F-actin capping protein alpha-1 subunit [Macaca mulatta] | 8 | gi\|109013601 (+2) | 33 kDa | 4 | Actin filament regulation |
| PREDICTED: chaperonin containing TCP1, subunit 6A isoform 6 [Pan troglodytes] | 5 | gi\|114613461 (+7) | 54 kDa | 4 | Chaperone |
| PREDICTED: similar to Protein S100-A11 (S100 calcium-binding protein A11) | 12 | gi\|109016674 (+3) | 61 kDa | 4 | Regulator of cell cycle progression |
| PREDICTED: similar to 14-3-3 protein gamma subtype; 14-3-3 gamma [Pan troglodytes] | 9 | gi\|114614147 (+12) | 36 kDa | 5 | Phosphoserine motif binding, signal transduction |
| PREDICTED: similar to procollagen, type VI, alpha 2 [Rattus norvegicus] | 2 | gi\|109509328 (+1) | 110 kDa | 4 | Structural protein |
| IGK protein [Bos taurus] | 9 | gi\|115545495 (+1) | 27 kDa | 4 | Immune response |
| Signal transducer and activator of transcription 1, 91kDa, isoform CRA_c [Homo sapiens] | 4 | gi\|119631258 (+12) | 82 kDa | 4 | Signal transduction |
| PREDICTED: similar to epsilon isoform of 14-3-3 protein isoform 5 [Pan troglodytes] | 9 | gi\|114665589 (+12) | 29 kDa | 5 | Phosphoserine motif binding, signal transduction |
| PREDICTED: chaperonin containing TCP1, subunit 7 isoform 4 [Macaca mulatta] | 5 | gi\|109103372 (+30) | 59 kDa | 4 | Chaperone |
| Apolipoprotein A-II [Bos taurus] | 12 | gi\|114052298 | 11 kDa | 4 | Lipid binding |
| PREDICTED: similar to Galectin-3 (Galactose-specific lectin 3) | 9 | gi\|109083717 | 27 kDa | 4 | Carbohydrate binding |
| PREDICTED: peroxiredoxin 6 [Macaca mulatta] | 9 | gi\|109019544 (+1) | 25 kDa | 4 | Redox regulation |
| Chain A, Crystal Structure of the Heterodimeric Complex of Human Rgs10 and Activated Gi Alpha 3 | 7 | gi\|119390147 (+3) | 37 kDa | 9 | Regulator of intracellular signaling |
| RecName: Full=Retinol-binding protein 4 | 10 | gi\|132403 (+4) | 21 kDa | 4 | Vitamin A binding protein |
| PREDICTED: similar to thrombospondin 1 precursor [Macaca mulatta] | 2 | gi\|109080672 (+4) | 157 kDa | 25 | Mediator of cell-cell or cell-matrix interactions |
| Claudin 1 [Homo sapiens] | 10 | gi\|10863887 (+6) | 23 kDa | 2 | Component of tight-junction cellular interactions |
| Nucleophosmin 1 isoform 1 [Homo sapiens] | 7 | gi\|10835063 (+54) | 33 kDa | 2 | Chaperonin for core histones, regulator of ARF/p53 pathway |
| Serpin peptidase inhibitor, clade G, member 1 [Bos taurus] | 4 | gi\|27807349 | 52 kDa | 2 | Protease inhibitor |
| Complement component 1, s subcomponent [Bos taurus] | 5 | gi\|115497210 (+1) | 77 kDa | 2 | Immune response |
| PREDICTED: similar to transient receptor potential cation channel, subfamily A, member 1 [Hydra magnipapillata] | 7 | gi\|221125287 | 167 kDa | 2 | Cation channel |
| PREDICTED: thioredoxin [Macaca mulatta] | 12 | gi\|109002483 | 12 kDa | 2 | Redox regulation |
| Chloride intracellular channel 1 [Oryctolagus cuniculus], | 8 | gi\|126722635 (+11) | 27 kDa | 2 | Anion transport |
| FCNB protein [Bos taurus] | 8 | gi\|133778314 (+1) | 43 kDa | 2 | Lectin binding |
| PREDICTED: Na+/K+ -ATPase beta 1 subunit isoform 4 [Macaca mulatta] | 6 | gi\|109019401 (+30) | 35 kDa | 2 | Cation channel |
| RecName: Full=Serpin A3-1 | 5 | gi\|160332365 (+2) | 46 kDa | 2 | Protease inhibitor |
| RuvB-like 1 (E. coli) [Homo sapiens] | 6 | gi\|119599729 (+16) | 44 kDa | 3 | ATP dependent helicase |
| 3-alpha hydroxysteroid dehydrogenase type IIb [Homo sapiens] | 8 | gi\|10765097 (+27) | 37 kDa | 3 | Metabolism |
| PREDICTED: enolase 3 [Pan troglodytes] | 6 | gi\|114665857 (+9) | 47 kDa | 8 | Glycogen storage |
| PREDICTED: similar to UDP-glucose dehydrogenase isoform 6 [Macaca mulatta] | 5 | gi\|109074025 (+16) | 55 kDa | 3 | Glycosamino-  glycan biosynthesis |
| GA20856 [Drosophila pseudoobscura pseudoobscura] | 10 | gi\|125809938 | 20 kDa | 4 | GTP binding |
| Regucalcin [Bos taurus] | 8 | gi\|27806809 | 33 kDa | 3 | Calcium binding protein, regulates calcium mediated signaling |
| Alpha-fetoprotein [Bos taurus] | 4 | gi\|77735479 | 69 kDa | 3 | Binds copper, nickel and fatty acids |
| PREDICTED: myosin IB [Pan troglodytes] | 3 | gi\|114582421 (+18) | 129 kDa | 3 | Motor protein |
| PREDICTED: chloride intracellular channel 4 isoform 3 [Macaca mulatta] | 9 | gi\|108999269 (+13) | 29 kDa | 3 | Anion channel |
| Unnamed protein product [Homo sapiens] | 4 | gi\|158255378 (+1) | 73 kDa | 3 | Unknown |
| Chaperonin GroEL [Agrobacterium radiobacter K84] | 5 | gi\|222085003 | 58 kDa | 3 | Chaperone |
| PREDICTED: similar to CTL2 protein [Macaca mulatta] | 4 | gi\|109123432 (+16) | 75 kDa | 3 | Chloride transport |
| Adiponectin | 9 | gi\|114158576 | 26 kDa | 3 | TNF alpha antagonist, regulates c-AMP dependent signaling |
| Proliferating cell nuclear antigen [Rattus norvegicus] | 8 | gi\|11693142 (+15) | 29 kDa | 3 | DNA polymerase delta accessory protein; Regulation of DNA replication |
| PREDICTED: similar to myosin IC [Macaca mulatta] | 3 | gi\|109112694 | 107 kDa | 3 | Motor protein |
| PREDICTED: similar to 60S ribosomal protein L12 [Monodelphis domestica] | 10 | gi\|126297606 (+1) | 32 kDa | 3 | Component of 60S ribosome |
| Coagulation factor XIII, B polypeptide [Bos taurus] | 4 | gi\|84370091 | 75 kDa | 3 | Coagulation |
| PREDICTED: similar to Calpain small subunit 1 (CSS1) | 9 | gi\|109124450 (+10) | 28 kDa | 3 | Protease |
| PREDICTED: eukaryotic translation elongation factor 2 [Macaca mulatta] | 4 | gi\|109122950 (+13) | 155 kDa | 3 | Regulation of translation |
| PREDICTED: similar to N-myc downstream regulated gene 1 isoform 5 [Macaca mulatta] | 7 | gi\|109087525 (+2) | 43 kDa | 3 | Signaling protein involved in stress response |
| PREDICTED: similar to Heterogeneous nuclear ribonucleoprotein D0 (hnRNP D0) | 7 | gi\|109074343 (+23) | 31 kDa | 3 | RNA binding protein |
| PREDICTED: actinin, alpha 1 isoform 1 [Pan troglodytes] | 3 | gi\|114653622 (+31) | 106 kDa | 8 | F-actin crosslinking protein |
| ADP-ribosylation factor 5 (predicted) [Callithrix jacchus] | 10 | gi\|167427284 (+4) | 19 kDa | 8 | ADP-ribosyltransferase involved in protein trafficking |
| PREDICTED: GDP dissociation inhibitor 2 isoform 5 [Macaca mulatta] | 6 | gi\|109088117 (+3) | 51 kDa | 3 | Regulator of GDP/GTP exchange of Rab proteins |
| PREDICTED: similar to transmembrane emp24 domain-containing protein 10 precursor | 10 | gi\|109084346 (+19) | 37 kDa | 3 | Type I membrane protein involved in vesicular trafficking |
| Hypothetical protein LOC100144939 [Xenopus (Silurana) tropicalis] | 9 | gi\|187607812 | 29 kDa | 3 | Unknown |
| PREDICTED: similar to serine hydroxymethyltransferase 1 (soluble) isoform 1 isoform 1 [Macaca mulatta] | 6 | gi\|109113536 (+2) | 53 kDa | 3 | Catalyzes the inter-conversion of serine and glycine |
| Glutathione S-transferase P [Macaca mulatta] | 9 | gi\|111185949 | 23 kDa | 2 | Catalyzes the conjugation of glutathione to electrophilic molecules |
| PREDICTED: similar to chaperonin containing TCP1, subunit 3 isoform a isoform 4 [Macaca mulatta] | 5 | gi\|109017347 (+37) | 61 kDa | 2 | Chaperone |
| Unnamed protein product [Mus musculus] | 6 | gi\|74212109 | 50 kDa | 13 | Unknown |
| PREDICTED: guanine  nucleotide binding protein (G protein), alpha inhibiting activity polypeptide 2 isoform 1 [Macaca mulatta] | 7 | gi\|109039529 (+8) | 40 kDa | 7 | Regulates G-protein mediated signaling |
| Elongation factor tu [Mycoplasma phocidae] | 7 | gi\|148616223 (+4) | 42 kDa | 2 | Regulates translation |
| Phosphofructokinase, platelet [Homo sapiens] | 4 | gi\|11321601 (+5) | 86 kDa | 2 | Metabolism |
| ADP-ribosylation factor-like 1 [Rattus norvegicus] | 10 | gi\|11693150 (+21) | 20 kDa | 3 | GTP-binding protein that plays a role in vesicular membrane trafficking |
| PREDICTED: transferrin receptor isoform 2 [Macaca mulatta] | 4 | gi\|109054368 (+1) | 85 kDa | 2 | Mediates cellular uptake of transferrin-bound iron |
| PREDICTED: glutamyl-prolyl tRNA synthetase isoform 6 [Pan troglodytes] | 5 | gi\|114572697 (+10) | 171 kDa | 2 | Catalyzes the aminoacylation of glutamic acid and proline tRNA |
| PREDICTED: lamin A/C [Macaca mulatta] | 9 | gi\|109017515 (+43) | 81 kDa | 2 | Component of the nuclear lamina |
| Chain A, Crystal Structure of Nore1a in Complex with Ras | 10 | gi\|194320091 (+1) | 19 kDa | 4 | Regulator of lymphocyte adhesion |
| Actin-related protein 2/3 complex subunit 4 [Scophthalmus maximus] | 10 | gi\|116488106 (+32) | 19 kDa | 2 | Regulator of actin polymerization |
| Chain A, Macrophage Migration Inhibitory Factor (Mif) Complexed with Inhibitor. | 12 | gi\|13399777 (+5) | 13 kDa | 2 | Pro-inflammatory cytokine |
| PREDICTED: hypothetical protein [Gallus gallus] | 11 | gi\|118099214 (+6) | 14 kDa | 2 | Unknown |
| PREDICTED: similar to cell division cycle 42 isoform 2 [Macaca mulatta] | 10 | gi\|109070989 (+77) | 21 kDa | 2 | Cell cycle regulation |
| 50S ribosomal protein L7/L12 [Mesorhizobium sp. BNC1], | 12 | gi\|110634170 | 13 kDa | 2 | 50S ribosome component |
| PREDICTED: similar to vesicle amine transport protein 1 [Macaca mulatta] | 6 | gi\|109115559 (+22) | 43 kDa | 2 | Transport protein involved in vesicle transport |
| Capping protein (actin filament) muscle Z-line, beta, isoform CRA_b [Homo sapiens] | 8 | gi\|119615295 (+24) | 30 kDa | 2 | Regulator of actin polymerization |
| Cystatin C [Macaca mulatta] | 11 | gi\|74136407 | 16 kDa | 2 | Cysteine protease inhibitor |
| PREDICTED: similar to Interleukin enhancer binding factor 2, 45kDa [Gallus gallus] | 7 | gi\|118120593 (+14) | 39 kDa | 2 | Transcription factor required for interleukin 2 expression |
| PREDICTED: similar to proteasome beta 5 subunit [Macaca mulatta] | 10 | gi\|109082916 (+33) | 28 kDa | 2 | Proteasome component |
| PREDICTED: similar to claudin 4 isoform 1 [Macaca mulatta] | 10 | gi\|109066321 (+4) | 22 kDa | 2 | Role in obliteration of extracellular tight junctions |
| PREDICTED: similar to carbonyl reductase 3 [Macaca mulatta] | 8 | gi\|109065366 (+6) | 31 kDa | 2 | Metabolic enzyme that reduces carbonyls to their corresponding alcohols |
| PREDICTED: eukaryotic translation initiation factor 3, subunit 9 eta, 116kDa [Macaca mulatta] | 3 | gi\|109065848 | 92 kDa | 2 | Regulation of translation |
| ITGA2B protein [Bos taurus] | 3 | gi\|151556350 | 114 kDa | 2 | Receptor for fibronectin, fibrinogen, plasminogen, prothrombin, thrombospondin and vitronectin |
| PREDICTED: eukaryotic translation initiation factor 3 subunit 6 interacting protein isoform 2 [Macaca mulatta] | 5 | gi\|109094135 (+25) | 68 kDa | 2 | Regulation of translation |
| PREDICTED: similar to NCK-associated protein 1 isoform 2 [Macaca mulatta] | 3 | gi\|109100376 (+33) | 150 kDa | 2 | Component of lamellipodial complex that controls Rac-dependent actin remodeling |
| PREDICTED: similar to proteasome subunit alpha type 6 | 9 | gi\|109083329 (+34) | 22 kDa | 2 | Proteasome component |
| CD59 antigen p18-20 allele B [Macaca fascicularis] | 10 | gi\|19716068 (+2) | 14 kDa | 2 | Inhibitor of the complement membrane attack complex (MAC) |
| PREDICTED: similar to solute carrier family 4 (anion exchanger), member 4 [Macaca mulatta] | 3 | gi\|109074556 (+63) | 121 kDa | 2 | Plasma membrane anion exchange protein |
| PREDICTED: similar to programmed cell death 6-interacting protein | 4 | gi\|109485522 (+28) | 97 kDa | 2 | Activator of apoptosis |
| PREDICTED: protein-L-isoaspartate (D-aspartate) O-methyltransferase isoform 2 [Macaca mulatta] | 9 | gi\|109072407 (+20) | 27 kDa | 2 | Metabolic enzyme that catalyzes the methyl esterification of L-isoaspartyl and D-aspartyl residues |
| Chain A, Crystal Structure of Human Dipeptidyl Peptidase Iv (Dppiv) Complexed with Cyanopyrrolidine (C5-Pro-Pro) Inhibitor 21ac, | 3 | gi\|110590190 (+22) | 84 kDa | 2 | Protease |
| Chain A, Crystal Structure of the P115rhogef Rgrgs Domain in a Complex with Galpha(13) | 7 | gi\|60593482 | 40 kDa | 6 | Regulates RhoA GTPase |
| Cystatin C [Bos taurus] | 12 | gi\|27806675 | 16 kDa | 2 | Cysteine protease inhibitor |
| Rab7 [Mus musculus] | 10 | gi\|1050551 (+24) | 24 kDa | 2 | Involved in acidification of phagosomes |
| PREDICTED: similar to programmed cell death protein 6 (Probable calcium-binding protein ALG-2) (PMP41) (ALG-257) [Macaca mulatta] | 10 | gi\|109076614 (+26) | 20 kDa | 2 | Activator of apoptosis |
| PREDICTED: peptidylprolyl isomerase B [Pan troglodytes] | 10 | gi\|114657576 (+6) | 24 kDa | 2 | Protein folding |
| PREDICTED: hypothetical protein [Ornithorhynchus anatinus] | 10 | gi\|149637213 (+1) | 21 kDa | 7 | Unknown |
| Translin [Rattus norvegicus] | 9 | gi\|11120712 (+20) | 26 kDa | 2 | DNA binding protein involved in recognition of double strand breaks |
| PREDICTED: methylenetetrahydrofolate dehydrogenase 1 isoform 1 [Macaca mulatta] | 3 | gi\|109083920 (+8) | 3104 kDa | 2 | Metabolism |
| Chain A, Ca2+-Binding Mimicry in the Crystal Structure of the Eu3+- Bound Mutant Human Macrophage Capping Protein Cap G | 7 | gi\|21730367 (+1) | 39 kDa | 2 | Regulation of actin polymerization |
| Superoxide dismutase 1, soluble [Macaca mulatta] | 11 | gi\|74136167 (+1) | 16 kDa | 2 | Redox regulation |
| PREDICTED: similar to tubulin-specific chaperone a [Macaca mulatta] | 11 | gi\|109004770 | 13 kDa | 2 | Chaperone of Tubulin |
| PREDICTED: copine III [Macaca mulatta] | 5 | gi\|109086863 (+5) | 69 kDa | 2 | Regulation of trafficking events occurring at the plasma membrane |
| Unnamed protein product [Homo sapiens] | 4 | gi\|221041684 (+6) | 44 kDa | 2 | Unknown |
| PREDICTED: coagulation factor X [Bos taurus] | 6 | gi\|119904982 (+3) | 55 kDa | 2 | Coagulation |
| PREDICTED: isocitrate dehydrogenase 1 (NADP+), soluble isoform 1 [Macaca mulatta] | 7 | gi\|109100767 (+59) | 47 kDa | 2 | Metabolism |
| Glutathione peroxidase 3 (plasma) [Bos taurus] | 10 | gi\|151554274 (+2) | 26 kDa | 2 | Redox regulation |
| PREDICTED: similar to destrin [Pan troglodytes] | 11 | gi\|114681108 (+15) | 28 kDa | 2 | Promotes actin depolymerization |
| Apolipoprotein E | 8 | gi\|114040 (+3) | 36 kDa | 4 | Mediates binding and internalization of lipoprotein particles |
| PREDICTED: similar to phosphoserine aminotransferase isoform 1 isoform 2 [Macaca mulatta] | 7 | gi\|109111852 (+1) | 40 kDa | 2 | Synthetic enzyme of 3-phosphohydroxy-pyruvate to phosphoserine and of 3-hydroxy-2-oxo-4 phosphonooxy-butanoate to phosphohydroxy-threonine |
| Phasin family protein [Sinorhizobium medicae WSM419] | 12 | gi\|150396334 (+1) | 13 kDa | 2 | Surface protein involved in Polyhydroxyal-kanoatestorage |
| PREDICTED: similar to synaptogyrin 2 isoform 3 [Macaca mulatta] | 9 | gi\|109118469 (+1) | 25 kDa | 2 | Integral membrane protein that may regulate membrane traffic |
| PREDICTED: hypothetical protein [Pan troglodytes] | 9 | gi\|114614145 (+7) | 21 kDa | 2 | Unknown |
| Serpin peptidase inhibitor, clade F | 6 | gi\|213021132 (+1) | 46 kDa | 2 | Protease inhibitor |
| Enolase 1 [Bos taurus] | 6 | gi\|87196501 | 47 kDa | 12 | Metabolism, transcription factor |
|  |  |  |  |  |  |
| PREDICTED: similar to creatine kinase, brain [Macaca mulatta] | 7 | gi\|109085011 (+20) | 30 kDa | 2 | Metabolism |
| PREDICTED: similar to 60S ribosomal protein L22 (Heparin-binding protein HBp15) isoform 1 [Macaca mulatta] | 11 | gi\|108996179 (+17) | 12 kDa | 2 | Ribosomal component |
| PREDICTED: vinculin isoform 1 [Pan troglodytes] | 3 | gi\|114631299 (+22) | 115 kDa | 2 | Actin binding protein involved in cell adhesion |
| Mannan-binding lectin serine protease 1 [Bos taurus] | 5 | gi\|116004151 | 81 kDa | 2 | Protease |
| DNA-binding protein [Homo sapiens] | 11 | gi\|1066080 (+20) | 19 kDa | 2 | DNA binding protein |
| PREDICTED: coatomer protein complex, subunit beta isoform 1 [Pan troglodytes] | 3 | gi\|114636300 (+16) | 107 kDa | 2 | Subunit of the coatomer complex required for budding from Golgi membranes |

**Table S2.** Proteins identified in native complex 1

| **Protein Name** | **AccessionNumber** | **Protein MWT** | **Total Peptide Count*** | **Xcorr** | **Delta Cn** | **Molecular Function** |
| --- | --- | --- | --- | --- | --- | --- |
| Histone H4 | P62805 | 11367 | 3 | 3.7637  3.9087  3.5608 | 0.3826  0.1523  0.4516 | Chromatin, DNA binding |
| MP12 nucleocapsid protein N | P21700 | 27431 | 3 | 2.9688  3.5431  2.7221 | 0.3075  0.2579  0.5502 | Viral RNA binding |
| Hemoglobin alpha subunit | P69905 | 15258 | 2 | 3.251 | 0.3109 | Oxygen transport |
| Hemoglobin beta subunit | P68871 | 15998 | 2 | 2.8083 | 0.3592 | Oxygen transport |
| Laminin subunit gamma-1 | P11047 | 177607 | 2 | 4.1871  2.9179 | 0.4855  0.5606 | Extracellular matrix |
| Actin, cytoplasmic 1 | P60709 | 41737 | 1 | 2.6364 | 0.4891 | Cytoskeleton, ATP binding |
| Alpha-enolase | P06733 | 47169 | 1 | 3.5197 | 0.6155 | Metabolism, transcription factor |
| ATP-binding cassette sub-family B member 7 | O75027 | 82641 | 1 | 2.3912 | 0.1705 | ATPase coupled transmembrane transport |
| Collectin-12 | Q5KU26 | 81525 | 1 | 2.803 | 0.2341 | Scavenger receptor, sugar binding |
| Crumbs homolog 1 | P82279 | 154183 | 1 | 2.4584 | 0.1356 | Calcium binding |
| Elongation factor 1-alpha 1 | P68104 | 50141 | 1 | 2.4174 | 0.2837 | Translation elongation, GTPase |
| Glyceraldehyde-3-phosphate dehydrogenase | P04406 | 36053 | 1 | 4.1076 | 0.5566 | Metabolism |
| HSP 90-beta | P08238 | 83264 | 1 | 3.4115 | 0.5278 | Chaperone, ATP binding |
| Histone H3.1t | Q16695 | 15508 | 1 | 2.7189 | 0.211 | Chromatin, DNA binding |
| Ig kappa chain V-III region NG9 | P01621 | 10729 | 1 | 2.5825 | 0.3452 | Cell adhesion |
| Inter-alpha-trypsin inhibitor heavy chain H2 | P19823 | 106436 | 1 | 2.5039 | 0.5459 | Peptidase Inhibition |
| Nance-Horan syndrome protein | Q6T4R5 | 176700 | 1 | 3.0102 | 0.1953 | Unknown |
| Protocadherin-17 | O14917 | 126229 | 1 | 2.9727 | 0.1 | Cell adhesion |
| SP110 nuclear body protein, isoform CRA_a | Q53TG2 | 78495 | 1 | 2.1291 | 0.1147 | DNA binding, regulation of transcription |
| RNA-binding motif protein, Y chromosome, family member F/J | Q15415 | 55728 | 1 | 2.5444 | 0.1424 | RNA binding |
| T-complex protein 1 subunit delta | P50991 | 57924 | 1 | 2.5293 | 0.331 | Chaperone, ATP binding |
| T-complex protein 1 subunit zeta | P40227 | 58024 | 1 | 3.8165 | 0.5266 | Chaperone, ATP binding |
| UPF0587 protein C1orf123 | Q9NWV4 | 18048 | 1 | 2.9661 | 0.1482 | Unknown |
| Zinc finger protein 420 | Q8TAQ5 | 80247 | 1 | 2.383 | 0.1991 | DNA binding, regulation of transcription |

*For each unique peptide within the indicated total peptide count, the Xcorr and Delat Cn values are listed in the table.

**Table S3.** Proteins identified in native complex 2

| **Protein Name** | **Accession Number** | **Protein MWT** | **Total Peptide Count*** | **Xcorr** | **Delta Cn** | **Molecular Function** |
| --- | --- | --- | --- | --- | --- | --- |
| MP12 Nucleo- protein N | P21700 | 27431 | 3 | 2.8532  3.7482  3.0552 | 0.5201  0.3016  0.2273 | Viral RNA binding |
| Pyruvate kinase isozymes M1/M2 | P14618 | 57937 | 3 | 3.0415  3.4107 | 0.4902  0.3881 | Metabolism |
| Serum albumin | P02768 | 69367 | 3 | 3.4293  3.5597  2.2724 | 0.5653  0.409  0.2531 | Carrier protein |
| Alpha-enolase | P06733 | 47169 | 2 | 3.0837  4.4085 | 0.443  0.6706 | Metabolism, transcription factor |
| Fibronectin | P02751 | 262625 | 2 | 4.0893 | 0.5667 | Extracellular matrix, cell adhesion |
| Hemoglobin alpha subunit | P69905 | 15258 | 2 | 3.4911 | 0.417 | Oxygen transport |
| Hemoglobin beta subunit | P68871 | 15998 | 2 | 2.5145  2.795 | 0.2665  0.2961 | Oxygen transport |
| Plectin-1 | Q15149 | 78878 | 2 | 4.1605  2.3178 | 0.5195  0.2296 | Actin binding, cytoskeleton |
| 39S ribosomal protein L40 | Q9NQ50 | 24490 | 1 | 2.3401 | 0.2641 | Mitochondrial protein synthesis |
| 78 kDa glucose-regulated protein | P11021 | 72333 | 1 | 3.3944 | 0.5012 | Chaperone, ATP binding |
| Actin, cytoplasmic 1 | P60709 | 41737 | 1 | 2.5757 | 0.4205 | Cytoskeleton, ATP binding |
| Alpha-1 type XI collagen isoform C preprotein variant | Q59HB5 | 99966 | 1 | 2.3171 | 0.1199 | Extracellular matrix  component |
| Alpha-2-macroglobulin | P01023 | 163278 | 1 | 3.0836 | 0.3876 | Protease inhibition |
| Alpha-actinin-1 | P12814 | 103058 | 1 | 3.3778 | 0.5135 | Actin filament binding, actin filament bundle formation |
| Annexin A2 | P07355 | 38604 | 1 | 3.8954 | 0.4312 | Phospholipase inhibitor, cytoskeleton binding |
| Beta-1,3-glucosyltransferase | Q6Y288 | 56564 | 1 | 3.0032 | 0.3319 | Phosphatidyl-choline-sterol- O-acyltransferase activity, Lipid metabolism |
| Erythrocyte band 7 integral membrane protein | P27105 | 31731 | 1 | 4.2267 | 0.6455 | Protein homo- oligomerization |
| Filamin-A | P21333 | 280739 | 1 | 2.5378 | 0.3158 | Actin cytoskeleton organization |
| Galectin-8 | O00214 | 35672 | 1 | 2.5918 | 0.1436 | Sugar binding |
| Glypican-4 | O75487 | 62412 | 1 | 3.662 | 0.4679 | Extracellular matrix |
| Heat shock protein 90-Beta | P08238 | 83264 | 1 | 2.694 | 0.3546 | Chaperone, ATP binding |
| Histone H2A type 1-B/E | P04908 | 14135 | 1 | 2.777 | 0.3227 | Chromatin, DNA binding |
| Histone H3.1t | Q16695 | 15508 | 1 | 2.3487 | 0.2676 | Chromatin, DNA binding |
| Histone H4 | P62805 | 11367 | 1 | 3.4629 | 0.5005 | Chromatin, DNAbinding |
| Homeobox protein Hox-A5 | P20719 | 29345 | 1 | 2.8314 | 0.1458 | Transcription factor |
| Ig alpha-1 chain C region | P01876 | 37655 | 1 | 3.3025 | 0.4962 | Protein binding |
| IQ motif and SEC7 domain-containing protein 1 | Q6DN90 | 108314 | 1 | 4.6669 | 0.3421 | ARF guanyl-nucleotide exchange |
| MORC family CW-type zinc finger protein 2 | Q9Y6X9 | 117823 | 1 | 2.8703 | 0.125 | ATP binding, zinc binding |
| Polyadenylate-binding protein 1 | P11940 | 70671 | 1 | 2.6177 | 0.1216 | RNA binding, translation activation |
| Polymeric immunoglobulin receptor | P01833 | 83284 | 1 | 3.4062 | 0.3711 | Immuno-globulin transport |
| Pregnancy zone protein | P20742 | 163832 | 1 | 3.1504 | 0.4925 | Protease inhibition |
| Pregnancy-specific beta-1-glycoprotein | Q15238 | 37680 | 1 | 2.3693 | 0.1347 | Antigen |
| Protein FAM70A | Q5J4V8 | 2121 | 1 | 2.5074 | 0.1057 | Unknown |
| Prothrombin | P00734 | 70037 | 1 | 4.117 | 0.5626 | Caspase activation, apoptosis, platelet activation |
| Putative uncharacterized protein | Q9GZZ5 | 14868 | 1 | 2.6953 | 0.1339 | Unknown |
| Regulating synaptic membrane Exocytosis protein 1 | Q86UR5 | 189073 | 1 | 2.4526 | 0.1188 | Neuro-transmitter release, Zinc binding |
| Talin-1 | Q9Y490 | 269767 | 1 | 3.8462 | 0.3465 | Anchoring cytoskeleton to plasma membrane |
| T-cell acute lymphocytic leukemia protein 1 | P17542 | 34271 | 1 | 2.6886 | 0.2487 | DNA binding, regulation of transcription |
| T-complex protein 1 subunit delta | P50991 | 57924 | 1 | 2.6265 | 0.3631 | Chaperone, ATP binding |
| Teashirt homolog 3 | Q63HK5 | 118566 | 1 | 2.6356 | 0.1032 | Transcription factor |
| Teneurin-3 | Q9P273 | 300950 | 1 | 3.0057 | 0.2916 | Unknown |
| Transcription elongation factor A protein-like 2 | Q9H3H9 | 25850 | 1 | 3.4406 | 0.1901 | Regulation of transcription |
| Transitional endoplasmic reticulum (TER) ATPase | P55072 | 89322 | 1 | 3.3678 | 0.5268 | ATPase activity, ER associated stress response |
| Transmembrane protein TTMA | A6NKL6 | 63928 | 1 | 2.3837 | 0.1195 | Unknown |
| Uncharacterized protein C17orf85 | Q53F19 | 70593 | 1 | 2.4755 | 0.1282 | Unknown |
| Zinc finger CCHC domain protein 2 | Q9C0B9 | 125936 | 1 | 2.3832 | 0.1433 | Nucleic acid binding |

*For each unique peptide within the indicated total peptide count, the Xcorr and Delta Cn values are listed in the table.

**Table S4**. Proteins identified in native complexes 3 and 4

| **Protein**  **Name** | **Accession Number** | **Protein MWT** | **Total Peptide Count*** | | X**Xcorr** | **Delta Cn** | **Molecular Function** |
| --- | --- | --- | --- | --- | --- | --- | --- |
| Caspase-2 (cmplx 3) | P42575 | 50685 | 1 | 2.5488 | | 0.5636 | Regulation of apoptosis, protease activity |
| Integrin alpha-3 | P26006 | 118698 | 10 | 3.031  3.6219  4.0927  2.6636  2.6163  2.8052  3.3059  2.4588 | | 0.2874  0.5796  0.5039  0.3325  0.3989  0.4237  0.4162  0.3992 | Metal ion binding, cell adhesion |
| Integrin beta-1 | P05556 | 88465 | 10 | 2.326  3.418  2.1627  2.1328  3.4261  2.5133  3.2015 | | 0.126  0.4761  0.2435  0.384  0.4357  0.2628  0.437 | Metal ion binding, cell adhesion |
| Histone H4 | P62805 | 11367 | 2 | 3.6106  3.3256 | | 0.4527  0.402 | Chromatin, DNA binding |
| Integrin alpha-1 | P56199 | 130848 | 2 | 3.5623  3.6265 | | 0.4692  0.3871 | Metal ion binding, cell adhesion |
| Serum albumin | P02768 | 69367 | 2 | 3.4788  3.6308 | | 0.4778  0.4852 | Carrier protein |
| Sodium/potassium-transporting ATPase subunit alpha-1 | P05023 | 112896 | 2 | 2.751  4.1656 | | 0.4205  0.4803 | ATP driven ion transport |
| 78 kDa glucose-regulated protein | P11021 | 72333 | 1 | 3.2886 | | 0.4727 | Chaperone, ATP binding |
| Abhydrolase domain-containing protein 8 | Q96I13 | 47331 | 1 | 2.6831 | | 0.1466 | Hydrolase activity |
| Alpha-actinin-4 | O43707 | 104854 | 1 | 3.066 | | 0.5366 | Actin filament binding, actin filament bundle formation |
| Alpha-enolase | P06733 | 47169 | 1 | 3.2438 | | 0.3931 | Metabolism, transcription factor |
| Apolipoprotein A-1 | P02647 | 36778 | 1 | 2.326 7 | | 0.4855 | Lipid binding |
| Arachidonate 5- lipoxygenase | P09917 | 77983 | 1 | 2.5651 | | 0.2289 | Lipoxygenae, oxido-reductase activity |
| Aspartoacylase-2 | Q96HD9 | 35241 | 1 | 2.6356 | | 0.1382 | Aspartoa-cylase, hydrolase activity |
| Beta-1,3-glucosyltransferase | Q6Y288 | 56564 | 1 | 2.5837 | | 0.1705 | Glucosyl-transferase activity, carbohydrate metabolism |
| CD151 antigen | P48509 | 28295 | 1 | 3.5821 | | 0.2138 | Cell adhesion |
| Cystatin-A | P01040 | 11006 | 1 | 2.6459 | | 0.4635 | Protease inhibition |
| Ferritin heavy chain | P02794 | 21226 | 1 | 3.8551 | | 0.4619 | Iron transport |
| Filamin-C | Q14315 | 290959 | 1 | 3.8791 | | 0.4068 | Actin binding, cytoskeleton |
| Galectin-8 | O00214 | 35677 | 1 | 2.5645 | | 0.2113 | Sugar binding |
| Glyceraldehyde-3-phosphate dehydrogenase | P04406 | 36053 | 1 | 3.5399 | | 0.5773 | Metabolism |
| Glypican-4 | O75487 | 62412 | 1 | 3.6545 | | 0.5311 | Extracellular matrix |
| HBS1-like protein | Q9Y450 | 75473 | 1 | 2.8231 | | 0.3075 | Nucleotide binding, translation elongation factor |
| Heat shock protein HSP90-alpha | P07900 | 84660 | 1 | 3.0573 | | 0.4747 | Chaperone, ATP binding |
| Hemoglobin subunit alpha | P69905 | 15258 | 1 | 3.3639 | | 0.3259 | Oxygen transport |
| Hemoglobin subunit beta | P68871 | 15998 | 1 | 2.8722 | | 0.2556 | Oxygen transport |
| Histone H3.1t | Q16695 | 15508 | 1 | 2.4812 | | 0.2391 | Chromatin, DNA binding |
| MP12 Nucleoprotein N | P21700 | 27431 | 1 | 3.8636 | | 0.3108 | Viral RNA binding |
| Protein FAM171A1 | Q5VUB5 | 97854 | 1 | 2.4677 | | 0.1176 | Unknown |
| Putative uncharacterized protein FLJ43582 | Q6ZUL3 | 24736 | 1 | 2.3434 | | 0.1224 | Unknown |
| Rab11 family-interacting protein 2 | Q7L804 | 58279 | 1 | 2.9247 | | 0.1244 | Protein transport |
| Testis-specific gene 118 protein | Q1ED39 | 51589 | 1 | 2.7828 | | 0.1771 | Unknown |
| Transmembrane protein TTMA | A6NKL6 | 63928 | 1 | 2.9407 | | 0.2532 | Unknown |

*For each unique peptide within the indicated total peptide count, the Xcorr and Delta Cn values are listed in the table.

**Table S5.** Common proteins between purified RVFV virions and non-infected control sample obtained by cell lysis and subjected to the same purification procedure as virions side by side

| **Accession Number** | **Protein Description** |
| --- | --- |
|  |  |
| gi\|157954061 | Alpha-2-macroglobulin [Bos taurus] |
| gi\|59857769 | Inter-alpha (globulin) inhibitor H4 (plasma Kallikrein-sensitive glycoprotein) [Bos taurus] |
| gi\|1351907 | Serum albumin |
| gi\|109119169 | PREDICTED: similar to fatty acid synthase [Macaca mulatta] |
| gi\|124056491 | RecName: Full=Complement C3 |
| gi\|12667788 | Myosin, heavy polypeptide 9, non-muscle [Homo sapiens] |
| gi\|62460494 | Hemoglobin, gamma [Bos taurus] |
| gi\|109087706 | PREDICTED: similar to plectin 1 isoform 3 [Macaca mulatta] |
| gi\|148232266 | Fibulin 1 [Bos taurus] |
| gi\|245563 | Apolipoprotein A-I, apoA-1 [Bos=cattle, Friesian-Holstein male calves aged 2-4 weeks, Peptide, 247 aa] |
| gi\|27806941 | Serine proteinase inhibitor, clade A, member 1 [Bos taurus] |
| gi\|27806751 | Alpha-2-HS-glycoprotein [Bos taurus] |
| gi\|111305821 | Valosin-containing protein [Homo sapiens] |
| gi\|109081748 | PREDICTED: pyruvate kinase 3 isoform 9 [Macaca mulatta] |
| gi\|114577902 | PREDICTED: annexin IV isoform 5 [Pan troglodytes] |
| gi\|220141 | VLA-3 alpha subunit [Homo sapiens] |
| gi\|148238273 | Inter-alpha globulin inhibitor H2 polypeptide [Bos taurus] |
| gi\|1065361 | Chain A, Human Adp-Ribosylation Factor 1 Complexed with Gdp, Full Length Non-Myristoylated |
| gi\|135806 | RecName: Full=Prothrombin |
| gi\|78045497 | Vitronectin [Bos taurus] |
| gi\|110331845 | Inter-alpha (globulin) inhibitor H3 [Bos taurus] |
| gi\|78369364 | group-specific component (vitamin D binding protein) [Bos taurus] |
| gi\|114052298 | Apolipoprotein A-II [Bos taurus] |
| gi\|27807349 | Serpin peptidase inhibitor, clade G, member 1 [Bos taurus] |
| gi\|77735479 | Alpha-fetoprotein [Bos taurus] |
| gi\|74212109 | Unnamed protein product [Mus musculus] |
| gi\|109065848 | PREDICTED: eukaryotic translation initiation factor 3, subunit 9 eta, 116kDa [Macaca mulatta] |
